# Suicidal Ideation, Suicide Attempts, and Suicide Mortality in Cancer: An Overview of Systematic Reviews with Meta-Analysis

**DOI:** 10.3390/cancers17111788

**Published:** 2025-05-27

**Authors:** Javier Martinez-Calderon, Marta Infante-Cano, Javier Matias-Soto, Alejandro Galan-Mercant, Saul Pineda-Escobar, Olga Villar-Alises, Veronica Perez-Cabezas, Cristina Garcia-Muñoz, Juan-Carlos Hernandez-Rodriguez

**Affiliations:** 1 Instituto de Biomedicina de Sevilla-IBiS (Hospitales Universitarios Virgen del Rocío y Macarena, CSIC, Universidad de Sevilla), 41013 Sevilla, Spain; jmcalderon@us.es; 2 Departamento de Fisioterapia, Universidad de Sevilla, 41009 Sevilla, Spain; 3 CTS 1110: Uncertainty, Mindfulness, Self, and Spirituality (UMSS) Research Group, 41009 Andalusia, Spain; martainfante0@gmail.com (M.I.-C.); msjavi93@gmail.com (J.M.-S.); ccriss.g@gmail.com (C.G.-M.); 4 Cochrane Rehabilitation, Functioning and Disability, London W1G 0AN, UK; 5 Departamento Ciencias de la Salud y Biomédicas, Universidad Loyola Andalucía, 41704 Sevilla, Spain; 6 Department of Nursing and Physiotherapy, University of Cadiz, 11009 Cadiz, Spain; veronica.perezcabezas@uca.es; 7 Department of Physiotherapy, Faculty of Nursing, Physiotherapy and Podiatry, University of Seville, 41009 Seville, Spain; saulpinedaescobar@gmail.com (S.P.-E.); olgavillar711@gmail.com (O.V.-A.); 8 Dermatology Department, Virgen del Rocío University Hospital, 41013 Seville, Spain; j.carlos.her.rod@gmail.com; 9 CTS-1088: Enfermedades Inmunomediadas (IMIDS) Research Group, 41009 Andalusia, Spain

**Keywords:** cancer, incidence, prevalence, overview, suicide, systematic review

## Abstract

Background: this overview of systematic reviews with meta-analysis summarized the epidemiology of suicide in cancer, mainly focused on prevalence, incidence, and risk. Methods: the CINAHL (via EBSCOhost), Embase, PsycINFO (via ProQuest), and PubMed databases were searched from inception to February 2024. AMSTAR 2 evaluated the methodological quality of reviews. The degree of overlap between reviews was calculated. Results: Twelve systematic reviews with meta-analysis were included. The prevalence of suicidal ideation in prostate cancer was 9.85% (95%CI 7.31–12.70%). The relative risk of suicidal ideation in prostate cancer was 2.01 (95%CI: 1.52–2.64) in comparison to controls without this type of cancer during the first 12 months after diagnosis. Furthermore, the risk of suicidal ideation was higher among individuals with bladder cancer in comparison to people without this clinical condition (HR 1.90, 95%CI 1.29−2.81). The crude suicide mortality rate per 100,000 person-years in prostate cancer was 47.1 per 100,000 person-years (95%CI 39.85–54.96). Incidence suicide death per 100,000 person-years was higher in sarcoma (60.99 per 100,000 person-years, 95%CI 17.37–214.19), esophagus (87.71 per 100,000 person-years, 95%CI 27.42–280.54), and pancreas (75.39 per 100,000 person-years, 95%CI 41.80–135.97). Finally, the standardized mortality ratio by suicide was higher in the pancreas (SMR 6.42, 95%CI 1.60–25.76), bone and cartilage (SMR 9.59, 95%CI 1.54–59.77), and mesothelioma (SMR 13.07, 95%CI 1.61–105.80). Conclusions: overall, meta-analyses underlined the relevance of suicide mortality in different cancer sites and geographical regions. Some meta-analyses also found suicidal ideation may be important in prostate or bladder cancer.

## Simple Summary

This overview of systematic reviews with meta-analyses examined the epidemiology of suicide in cancer patients, focusing on prevalence, incidence, and risk across tumor types. Twelve reviews were included following a comprehensive database search and quality assessment using AMSTAR 2. The prevalence of suicidal ideation in prostate cancer was 9.85%, with a twofold increased risk compared to non-cancer controls during the first year post-diagnosis (RR 2.01). Individuals with bladder cancer also showed elevated risk (HR 1.90). Suicide mortality rates were highest in sarcoma (60.99 per 100,000 person-years), esophageal (87.71), and pancreatic cancers (75.39). The standardized mortality ratio (SMR) was notably elevated in pancreatic (SMR 6.42), bone and cartilage (SMR 9.59), and mesothelioma cases (SMR 13.07). These findings highlight a significant mental health burden in oncology, with specific cancer types presenting a disproportionately higher suicide risk. Targeted prevention strategies are warranted, particularly during early post-diagnosis periods and in high-risk tumor subgroups.

## 1. Introduction

Psychiatric disorders are common among patients with cancer [1]. Studies around the world have found that 35–40% of individuals with cancer may live with psychiatric comorbidities [2,3,4,5]. Depression and anxiety are probably the psychological factors more studied in oncology research and their global prevalence is high in different types of cancer (e.g., breast or digestive cancer) [6], Depression and anxiety may increase the risk of cancer-specific mortality and all-cause mortality [7], and both have been associated with suicidal trends [8,9]. 

Currently, there are discrepancies regarding the trend of suicide in cancer. For example, some studies developed in the United States found overall cancer-related suicide decreased from 1998 to 2018 [10], and declined in specific cancer sites (prostate cancer survivors) from 1975 to 2019 [11]. On the other hand, another study observed mortality rates by suicide may have been higher in North American cancer survivors than the general population between 1975 and 2020 [11]. The increase in suicide among individuals with cancer has been also detected in other geographical regions such as India, where trends in cancer-related suicide have increased during the last five years [12].

The epidemiology of suicide in cancer is a topic of substantial interest for clinicians and researchers and the number of original studies in this field is constantly increasing. Multiple factors may favor the risk of suicide such as gender, cancer sites, disease stage, pre-existing mental illness, prognosis, poverty, or settings (rural versus metropolitan areas), among others [13]. The constant growth of the number of observational studies in this field has caused multiple systematic reviews with meta-analyses that have been published in the last five years aiming to summarize available evidence considering the prevalence, the incidence, or the risk of suicide in different cancer sites and geographical regions [14,15,16,17,18]. 

For example, Ding et al. [14] synthesized studies evaluating the prevalence of depression and suicidal ideation among Chinese cancer patients and explored its potential associated factors. This systematic review analyzed more than 200 studies and found that national pooled prevalence rates particularly of suicidal ideation reached more than 20%. Heinrich et al. [16] summarized evidence on suicide mortality risk according to different factors such as cancer prognosis, disease stage, time since cancer diagnosis, gender, ethnicity, marital status, year of recruitment, and geographic region. This systematic review evaluated more than 22,000 participants and observed that suicide mortality among people with cancer may be higher than the general population. Hofmann et al. [17] synthesized previous cohort studies considering the role of suicide in lung cancer. This systematic review also found that suicide mortality may be higher specifically in people with lung cancer in comparison to the general population.

All these previous systematic reviews and others have underlined the relevance of studying and synthesizing the epidemiology of suicide in people with cancer. Therefore, the development of an overview of systematic reviews with meta-analysis may be adequate and timely to help oncology health professionals and oncology researchers know the trends considering the value of meta-analyses on this topic, know the methodological quality of these systematic reviews as well as showing if the conclusions of these systematic reviews follow or not the same direction. The objective of this overview of systematic reviews with meta-analysis was to summarize evidence on the prevalence, the incidence, and the risk of different types of suicide (e.g., suicidal ideation or suicide attempt) in people with cancer, focusing the results mainly on the epidemiology of suicide by cancer sites and geographical regions, when possible.

## 2. Materials and Methods

We followed the PRIOR statement [19] and the PRISMA statement for abstracts [20]. The review protocol was prospectively registered at Open Science Framework (https://doi.org/10.17605/OSF.IO/ZSDTA) (accessed on 12 February 2024).

### 2.1. Deviations from the Protocol

Some deviations of the review protocol were made to reach more direct conclusions. First, continent disparities were not explored. The included reviews may have missed some original studies that may provide important information to explore possible continent disparities in this field. Second, associated factors with suicide were not extracted since we considered this topic may be broad and deserve a specific overview of systematic reviews. Third, the degree of overlap between reviews was only calculated when at least two reviews evaluated the same type of suicide (e.g., suicide mortality). In addition, we also calculated the degree of overlap between reviews when at least two reviews analyzed the same cancer site (e.g., breast cancer) and suicide (e.g., suicidal ideation). Fourth, the World Health Organization Classification of tumors (https://tumourclassification.iarc.who.int/welcome/, accessed on 12 February 2024) was not applied. This allowed us to use the word “cancer” instead of “tumor” throughout the manuscript which may be helpful for clinicians and researchers. Fifth, we decided to develop global maps to show the pooled results by geographical regions (continents). This allowed us to depict a better visualization of these results which may be useful for readers.

### 2.2. Data Sources and Search Strategy

The CINAHL (via EBSCOhost), Embase, PsycINFO (via ProQuest), and PubMed databases were searched by one co-author (JMC) from inception to 12 February 2024. The following search filters were used when possible: the type of document and the language of publication. Overviews of systematic reviews and published review protocols were manually checked if they were retrieved by the electronic databases during the search strategies. An example of a search strategy, specifically in PubMed is shown here (neoplasms [mh] OR oncolo* [tiab] OR cancer* [tiab] OR metasta* [tiab]) AND (suicide [tiab] OR suicides [tiab] OR suicidal [tiab] OR parasuicide [tiab] OR parasuicides [tiab] OR fatal-attempt [tiab] OR fatal-attempts [tiab] OR euthanasia [tiab] OR assisted-death [tiab] OR assisted-deaths [tiab] OR hasten-death [tiab] OR hastened-death [tiab]) AND (review [title] OR meta-analysis [title] OR meta-review [title] OR meta-analytic-review [title] OR meta-analysis [title] OR meta-analyses [title] OR overview* [title] OR umbrella [title]), and the rest of the search strategies for each electronic database is shown in Supplementary File S1.

### 2.3. Eligibility Criteria

The eligibility criteria were built using the Patient, Exposure, Comparison, Outcome, Study design (PECOS) framework [21]. Only studies published in peer-reviewed journals and written in English or Spanish language were considered.

#### 2.3.1. Inclusion Criteria

P: People are diagnosed with any type of cancer. There were no restrictions in terms of clinical (e.g., cancer site, disease stage, or time since cancer diagnosis) or sociodemographic (e.g., age or sex/gender) characteristics.

E: Any type of suicide (e.g., ideation, attempt, or suicide death). Mortality by suicide was also considered.

C: Not applicable.

O: Pooled prevalence, incidence, mortality rates, and risk of any type of suicide. 

S: Systematic reviews with meta-analysis.

#### 2.3.2. Exclusion Criteria

[I] Reviews where meta-analyses combined studies evaluating cancer and studies analyzing other health conditions.

[II] Reviews where meta-analyses combined studies exploring distinct types of suicide (e.g., ideation, attempt, and suicide death).

[III] Reviews where meta-analyses explored risk or associated factors of suicide.

[IV] Reviews where meta-analyses examined interventions to modify suicide outcomes.

[V] Conference abstracts and proceedings.

[VI] Impossibility of accessing full text.

[VII] Systematic review protocols.

### 2.4. Study Selection

**Figure 1:**
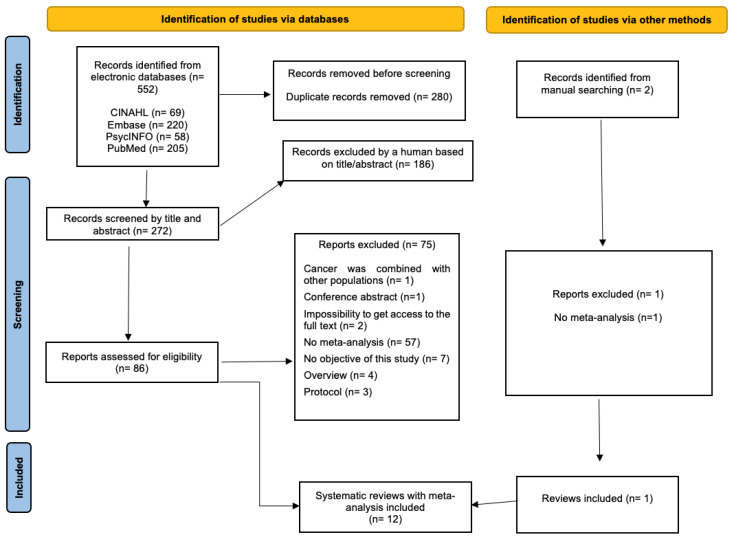
Flow diagram: study selection process.

Study selection was conducted by one co-author (JMC) using Zotero 6.0.36 Citation Management Software. All references were manually checked, and duplicates were manually removed. Afterward, titles and abstracts were read, and subsequently, full texts were evaluated if abstracts seemed eligible or if abstracts were unavailable. One study needed consensus with a second co-author (CGM), which was excluded [22]. Supplementary Files S3 and S4 and Figure 1 show the study selection process and the list of excluded studies and reasons for exclusion.

### 2.5. Methodological Quality Assessment

Four co-authors (JMS, MIC, OVA, and SPE) independently used AMSTAR 2 to assess the methodological quality of reviews [23]. JMS and SPE evaluated independently 50% of the included studies, whereas MIC and OVA assessed independently the rest of the included reviews. Disagreements between co-authors were resolved by consensus. The inter-rater agreement between JMS/SPE and MIC/OVA was calculated in percentages. For this, we considered the number of items co-authors assessed with the same score before pooling the results of their independent assessments. We adjusted some items of AMSTAR 2 to the requirements of epidemiological reviews. For items 1 ‘Did the research questions and inclusion criteria for the review include the components of PICO?’, and 8 ‘Did the review authors describe the included studies in sufficient detail?’, aspects related to ‘Intervention’ and ‘Comparator group’ were not applicable. AMSTAR 2 is composed of 16 items that can be rated as yes, partially yes, or no. Seven of these items are considered critical domains (items: 2, 4, 7, 9, 11, 13, 15), and an overall score is not recommended [23]. 

### 2.6. Data Extraction

Data extraction was conducted by one co-author (JMC). Consensus was needed with a second co-author (CGM) regarding some statistical analyses. Subgroup meta-analyses were extracted by cancer site or geographical region (continent), when possible. Corresponding authors were contacted to request additional information or clarify aspects associated with their methodology or reporting results. The following domains were extracted from each systematic review: first author and year of publication; if the systematic review performed meta-regression analyses, subgroup meta-analyses, and sensitivity analyses; the instrument to evaluate the risk of bias or the methodological quality of original research; the certainty of evidence assessment using the GRADE approach; population of interest (focusing on cancer site); outcome of interest; the number of original research and participants or interest; main findings (meta-analysis findings).

### 2.7. Degree of Overlap Between Reviews

The degree of overlap between meta-analyses of interest was calculated by one co-author (JMC) when at least two systematic reviews meta-analyzed the same type of suicide (e.g., suicidal mortality). When possible, we calculated the degree of overlap between meta-analyses by cancer site. The degree of overlap is calculated through the corrected covered area (CCA), which is the area covered after removing the primary studies the first time they are counted. Three steps are developed to calculate the CCA [24]. N is the total number of original studies (including duplicates) in the meta-analyses of interest (the sum of all checked boxes in the citation matrix). Furthermore, r is the number of original studies without accounting for duplicates. Finally, c is the number of systematic reviews included in the evidence matrix (k = 7). The overlap can be classified as slight (CCA 0–5%); moderate (CCA 6–10%); high (CCA 11–15%); or very high (CCA > 15%) [24]. To allow a visual comparison of the overlap findings a reviewer (CGM) performed a bar plot.

### 2.8. Data Synthesis

The results were summarized in the text by type of suicide (e.g., suicidal ideation) and cancer site (e.g., prostate cancer). Those reviews where pooled results were not shown by cancer site or geographical region were not reported in the text, but these meta-analyses were maintained in the table of characteristics of studies for transparency (Supplementary File S2). This decision was made to avoid misdirected conclusions due to the wide number of the included meta-analyses.

Pooled results by type of suicide and geographical region were reported in the text using global maps that underlined the continents that were explored. One co-author (CGM) used the Datawrapper app, developed by Datawrapper GmbH (https://app.datawrapper.de/select/map, accessed on 12 February 2024), to build the choropleth maps. CGM selected continents that were colored and made some manual annotations once global maps were generated. When more than two reviews reported the same type of suicide, continent, and epidemiological parameter (e.g., standardized mortality ratio), the different values from the included meta-analyses were selected. Grey color was used in global maps to depict the lack of data in some continents.

## 3. Results

A total of 552 references, including duplicates, were retrieved from electronic databases. Once duplicates were removed, 272 titles and abstracts were read. Afterward, 88 full texts, including two references manually found, were analyzed. Finally, 12 systematic reviews with meta-analysis were included [14,15,16,17,18,25,26,27,28,29,30,31]. See Supplementary Files S3 and S4 and Figure 1.

### 3.1. The Main Characteristics of the Included Reviews

Seven systematic reviews included meta-analyses by cancer sites [15,16,17,25,26,27,28]. One systematic review did not report information about whether multiple cancer sites or a specific cancer site were meta-analyzed [31]. The following cancer sites were explored: biliary system, bladder, bone and cartilage, breast, bronchus, central nervous system, cervix, colon, endometrium, esophagus, gastric, head and neck, hematological system, Hodgkin lymphoma, kidney, leukemias, liver, lung, melanoma, mesothelioma, non-Hodgkin lymphoma, ovary, pancreas, penis, prostate, rectum, sarcoma, skin, stomach, testicular, thyroid, trachea, urinary system, uterus, vulvar. In addition, seven systematic reviews developed subgroup meta-analyses by geographical region [14,16,17,25,26,27,28]. Mainly, systematic reviews evaluated the following continents: America, Asia, Europe, and Oceania. Most of the systematic reviews meta-analyzed suicide mortality [16,17,18,25,26,27,29,30,31]. Five systematic reviews also meta-analyzed suicidal ideation [14,15,18,26,28], whereas one systematic review meta-analyzed suicide attempt [18].

Methodologically, no systematic reviews used the GRADE approach to rate the certainty of evidence. Supplementary File S2 shows the main characteristics of the included studies and all pooled results regarding the included meta-analyses. We requested additional information or clarification via email for four studies [14,15,18,28], and the corresponding authors of Guo et al. 2018 and 2021 responded to our doubts [15,28]. The rest of the authors did not respond.

### 3.2. Results from Degree of Overlap Between Reviews

The overlap between reviews and meta-analyzing suicide mortality was high (CCA = 12%, k = 7). No overlap between reviews and meta-analyzing suicidal ideation was found (CCA = 0%, k = 3) (Supplementary Files S5 and S6). Two reviews meta-analyzing suicide mortality could not be included in the overlap. Heinrich et al. 2022 did not show some important references in their list of references [16]. Rafiei et al. 2023 did not report the references included in the meta-analysis of suicide death [18]. Two reviews meta-analyzing suicidal ideation could not be included in the overlap calculation. Ding et al. 2023 did not specify the references in the meta-analysis of interest [14]. Rafiei et al. 2023 were not included for the same reason [18]. Suicide attempts were only meta-analyzed by one review [18]. 

The overlap between reviews by cancer sites showed a very high overlap between meta-analyses evaluating prostate cancer and suicide mortality (CCA = 40%, k = 3) [25,26,28]. Reviews meta-analyzing suicide mortality and lung, bronchus, and/or trachea cancer also showed a very high overlap (CCA = 25%, k = 2) [17,25]. No overlap was found between reviews meta-analyzing prostate cancer and suicidal ideation (CCA = 0%, k = 2) [26,28] (Supplementary Files S7 and S8). Two reviews were not included in the overlap calculation because the references used in their meta-analyses by cancer sites were not specified [16,27]. 

### 3.3. Results from Methodological Quality Assessment

No reviews checked all items from AMSTAR 2 (Supplementary File S9). MIC and OVA independently assessed the first six studies in the table and the inter-rater agreement was 77%. JMS and SPE independently evaluated the six remaining studies, and the inter-rater agreement was 94.8%. Mainly, no reviews completely reported a list of excluded studies and reasons for exclusion (item 7). No reviews described completely the original studies in detail (item 8). In addition, no reviews reported sources of funding for the original studies (item 10), but most of the reviews reported information about their possible conflict of interests/funding (item 16). All reviews included the components of PECOS to build their eligibility criteria (item 1) and most of the reviews checked some items of AMSTAR 2 related to the development of meta-analysis (item 10) and the discussion and interpretation of the results (item 14). 

### 3.4. Suicidal Ideation by Cancer Site

Prevalence: the prevalence of suicidal ideation in prostate cancer was 9.85% (95%CI 7.31–12.70%) [26].

Risk: the relative risk of suicidal ideation in prostate cancer was 2.01 (95%CI: 1.52–2.64), I2 = 91.8% in comparison to individuals without this type of cancer during the first 12 months after diagnosis [28]. Similar results were found for bladder cancer, where the risk of suicidal ideation was higher among individuals with bladder cancer in comparison with those people without this clinical condition, hazard ratio of 1.90 (95%CI 1.29−2.81), I2 = 81.2% [15].

### 3.5. Suicide Mortality by Cancer Site

**Figure 2:**
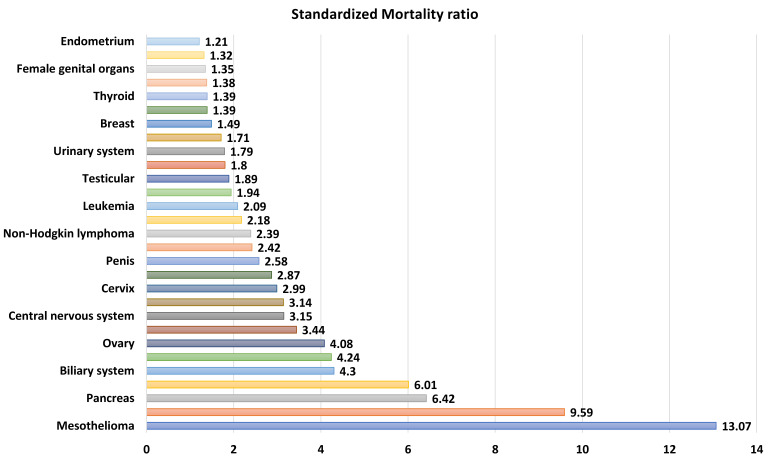
Pooled results of incidence of suicide death per 100,000 person-years for different cancer sites.

Incidence suicide death per 100,000 person-years (95%CI): From all sites, the esophagus has the highest incidence data with 87.71 per 100,000 person-years (27.42–280.54), while the uterus was the lowest with 6.19 per 100,000 person-years (4.41–8.70) [27]. Figure 2 and Supplementary File S2 show the incidence data from all cancer sites.

Crude suicide mortality rate per 100,000 person-years in prostate cancer was 47.1 per 100,000 person-years (95%CI 39.85–54.96) [26].

**Figure 3:**
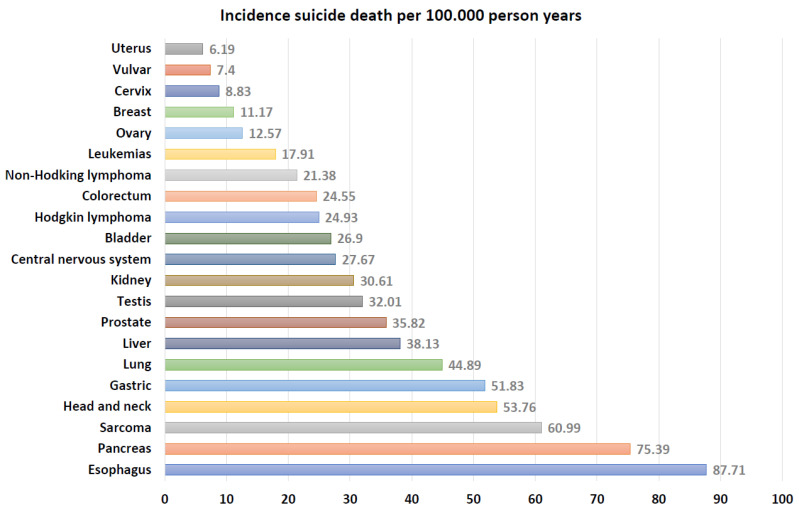
Pooled results of standardized mortality ratio for different cancer sites.

Standardized Mortality ratio (SMR 95%CI): from all sites, mesothelioma presented the highest SMR with 13.07 (1.61–105.80), while endometrium showed the lowest SMR with 1.21 (0.68–2.16) [16,17,25]. Figure 3 and Supplementary File S2 show the incidence data from all cancer sites.

### 3.6. Suicide by Geographical Regions

**Figure 4:**
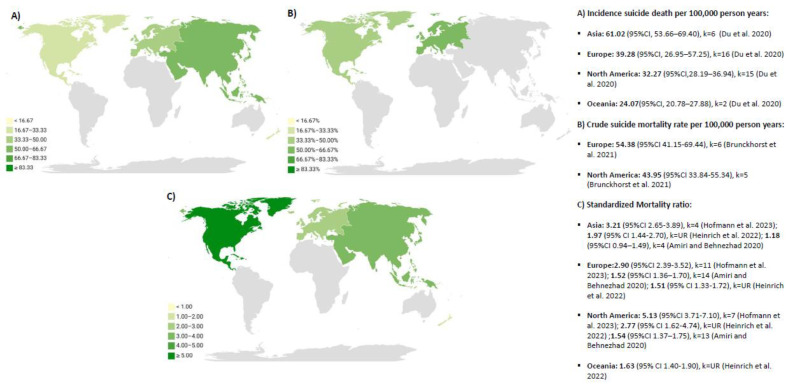
Continents distribution for incidence, crude suicide mortality rate, and standardized mortality ratio [16,17,25,26,27].

Figure 4 shows a global map reporting the meta-analyses of the incidence of suicide death per 100,000 person-years, crude suicide mortality rate per 100,000 person-years, and standardized mortality ratio by geographical regions [16,17,25,26,27]. In the case of North America, it was shown the highest value of standardized mortality ratio, but Europe and Asia presented the highest data for crude rates and incidence of suicide in cancer, respectively.

**Figure 5:**
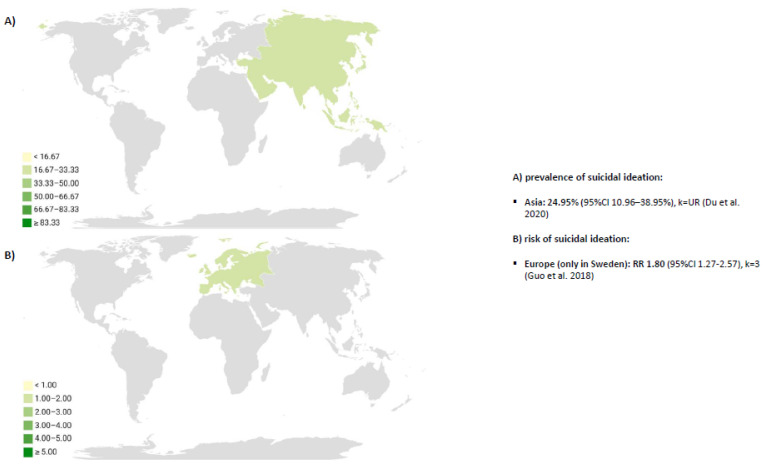
Continents distribution for prevalence suicidal ideation [18,27].

Figure 5 shows a global map reporting the meta-analyses of the prevalence of suicidal ideation, only studied in Asia (China) and the risk of suicidal ideation by geographical regions, only assessed in Europe [14,28]. No data were found for African countries for any of the outcomes of interest.

## 4. Discussion

This overview of systematic reviews with meta-analysis synthetizes the prevalence, mortality, incidence, and risk of suicide in cancer. Overall, the meta-analyses underlined the relevance of suicide mortality in different cancer sites. Some meta-analyses also found suicidal ideation may be important in prostate or bladder cancer. In terms of suicide mortality, North America showed the highest value of the standardized mortality ratio, but Europe and Asia presented the highest data for crude rates and incidence of suicide in cancer, respectively. Regarding suicidal ideation, the prevalence of suicidal ideation was only meta-analyzed in China, whereas the risk of suicidal ideation was only meta-analyzed in Europe. No data were found for African countries for any of the outcomes of interest.

### 4.1. Methodological Considerations

Suicide in patients with cancer has been estimated to be higher than the general population. Calati et al. [29] found that the standardized mortality ratio of suicide in cancer patients was 1.5 to 1.7-fold higher than in the general population after analyzing several review articles. Recently, important observational studies have also supported these results. Hunsberger et al. [30] evaluated 69,493 people with cervical cancer and found that the risk of suicide may be 8.8 times higher than the general population. Wang et al. [31] examined more than 50,000 individuals and observed the suicide rate in cancer was almost 3.5 times that of the general population. These are just a few examples that highlight the impact of cancer compared to the general population. In this context, the development of accurate subgroup analyses by cancer sites would help to achieve sounder conclusions. This could help to propose tailored strategies to reduce mortality, incidence, risk, and prevalence in patients depending on the cancer site. However, four out of twelve included reviews did not report the cancer site, which may affect the overall conclusion of this overview since this situation prevented us from including them in the main text [18,32,33,34].

Although this is not the scope of this overview, multiple factors may have affected the rates of prevalence, incidence, risk, and mortality found across the included meta-analyses. For example, some studies have underlined the role of cultural and ethnic factors in suicide outcomes among patients with cancer or the general population [35,36]. We observed suicide mortality, measured with the standardized mortality ratio, may be higher in the Americas (specifically in North America) in comparison to other continents. Although this fact may be related to major accessibility to firearms [37], the availability of firearms need not be the reason for higher rate of suicides in the US, and it may simply be a poor coping mechanism or an absence of family buffer. Future studies on this topic are highly relevant to detecting potential risk factors in people with cancer. In addition, it is important to note that data registries in developed countries (e.g., the United States) may be more accurate than those in low- and middle-income countries. Indeed, it is notable that none of the systematic reviews have reported data regarding South America or Africa.

Another important point is associated with the prognosis of patients with cancer. In this sense, systematic reviews have found cancer sites with poorer prognosis or recovery, such as sarcoma, pancreatic cancer, or mesothelioma, showed higher mortality or incidence of suicide. This fact is supported by previous evidence, underlying the relationship between the presence of cancer with poor prognosis and suicidal ideation, suicide attempts, and suicide mortality [38].

It is also important to note that some systematic reviews evaluated the number of suicide deaths and suicide attempts. Specifically, one systematic review [18] showed that 22,839 out of 67,169 patients died by suicide, which should help us strengthen health policies for people with cancer to try to prevent this enormous number of cases. Furthermore, it was important to highlight that the number of suicide attempts was over 40,000 cases, which could suggest that many people who attempt suicide do not ultimately die. Although we do not know the specific causes related to suicide attempts, future studies should investigate this issue.

Finally, none of the systematic reviews applied the GRADE approach to rate the certainty of evidence. The GRADE approach is based on essential domains that are key to understanding the certainty and strength of the meta-analyzed findings. Aspects such as the risk of bias, inconsistency, indirectness, imprecision, and publication bias are rated. Therefore, it is important that the authors of systematic reviews consider these factors when systematic reviews are performed. Although there is not a specific GRADE guideline for rating systematic reviews of epidemiological studies, using GRADE in these reviews may be recommendable [39]. We encourage authors of future systematic reviews in this field to apply it.

### 4.2. Clinical Implications

Suicide screening approaches and cognitive behavioral therapies or problem-solving therapies have been postulated as some of the possible strategies targeted to reduce suicide in this population. However, lack of training of the different health professionals, lack of access to mental health services, limited time of professionals with patients, and discomfort or fears of health professionals in confronting suicide have been found as barriers to the implementation of these strategies [40,41,42].

### 4.3. Future Research

If there are sufficient original studies, future research lines related to the synthesis of evidence may be associated with the risk of suicide attempts in different cancer sites and geographical regions. Future systematic reviews should also investigate the epidemiology of other types of suicide (e.g., assisted suicide) in cancer. Considering this line, it is also essential that future primary research and subsequent systematic reviews examine if physician-assisted suicides in countries where it is legal far exceed the suicide rates in non-cancer persons in that same geographical area. In addition, future overviews of systematic reviews or systematic reviews with meta-analyses on cancer should explore using subgroup meta-analyses the stratification of mortality risk by cancer stage and type of chemotherapy as they have a high impact on the results. All this information may help to develop new cancer epidemiology politics that help to reduce suicide rates worldwide. Methodologically, future systematic reviews with meta-analysis on this topic may aim to use the GRADE approach, which may help us better understand the possible presence of inconsistencies, indirectness, and imprecisions in the meta-analyzed results.

### 4.4. Limitations

This overview of systematic reviews with meta-analyses has some limitations that need to be acknowledged. First, thesis, dissertations, conference abstracts, and proceedings were not considered. Second, only systematic reviews with meta-analyses written in English or Spanish language were included. It may help to explain the total lack of information in continents such as Africa or countries such as Russia. Third, the absence of the GRADE approach in all systematic reviews could affect the certainty of our results. Fourth, the lack of subgroup meta-analyses in some included systematic reviews considering regions and cancer sites could limit the real status of the current state of the art. Fifth, only one co-author performed the study selection and data extraction process. This may have affected the methodological quality of the current overview. Finally, we relied entirely on secondary data, which prevented us from accessing primary study-level information. As a result, it was not possible to perform additional stratified analyses (e.g., by cancer type, gender, or geographic region), which may limit the generalizability of our conclusions.

## 5. Conclusions

Suicide in cancer is a topic of great interest for clinicians and researchers. Many systematic reviews have been published in the last three years considering the epidemiology of suicide in this clinical condition. Overall, the meta-analyses underlined the relevance of suicide mortality in different cancer sites. Some meta-analyses also found suicidal ideation may be important in prostate or bladder cancer. In terms of suicide mortality, North America showed the highest value of the standardized mortality ratio, but Europe and Asia presented the highest data for crude rates and incidence of suicide in cancer, respectively. Regarding suicidal ideation, the prevalence of suicidal ideation was only meta-analyzed in China, whereas the risk of suicidal ideation was only meta-analyzed in Europe. No data were found for African countries for any of the outcomes of interest.

## Data Availability

The study did not report any data.

## Supplementary Materials

The following supporting information can be downloaded at: https://www.mdpi.com/article/10.3390/cancers17111788/s1. Supplementary File S1: Search strategies; Supplementary File S2: Characteristics of SRs; Supplementary File S3: Flow Diagram; Supplementary File S4: Excluded studies list; Supplementary File S5: Overall Overlap; Supplementary File S6: Bar plot overall overlap; Supplementary File S7: Overlap Prostate Cancer; Supplementary File S8: Bar plot overlap cancer sites; Supplementary File S9: AMSTAR 2.
