# Supplementary material for: Suicidal Ideation, Suicide Attempts, and Suicide Mortality in Cancer: An Overview of Systematic Reviews with Meta-Analysis
Source: Cancers (Basel). 2025 May 27;17(11):1788. doi: 10.3390/cancers17111788 (PMC12153619; doi:10.3390/cancers17111788)
Supplement: Supplementary file 1 [file cancers-17-01788-s001.zip › Suppl File 1 Search strategies.pdf]

Supplementary file 1. Search Strategies in electronic databases.

CINAHL (date 12/02/2024)

(MH "Neoplasms+" OR AB (cancer* OR oncolo* OR metasta*)) AND AB (suicide OR suicides OR suicidal OR parasuicide OR parasuicides OR fatal-attempt OR fatal-attempts OR euthanasia OR assisted-death OR assisted-deaths OR hasten-death OR hastened-death) AND TI (review OR meta-analysis OR meta-review OR meta-analytic-review OR metaanalysis OR meta-analyses OR overview* OR umbrella)

Search modes - Boolean/Phrase.

Search filter: Type of document: scholarly publications, courses.

Search filter: Language of publication: English, Spanish.

Studies retrieved = 69

Embase (date 12/02/2024)

('malignant neoplasm'/exp OR 'oncology'/exp OR 'metastasis'/exp OR cancer*:ab,ti OR metasta*:ab,ti OR oncolo*:ab,ti) AND (suicide:ab,ti OR suicides:ab,ti OR suicidal:ab,ti OR parasuicide:ab,ti OR parasuicides:ab,ti OR 'fatal attempt':ab,ti OR 'fatal attempts':ab,ti OR euthanasia:ab,ti OR 'assisted death':ab,ti OR 'assisted deaths':ab,ti OR 'hasten death':ab,ti OR 'hastened death':ab,ti) AND (review:ti OR 'meta analysis':ti OR 'meta review':ti OR 'meta analytic review':ti OR metaanalysis:ti OR 'meta analyses':ti OR overview*:ti OR umbrella:ti)

Search filter: Type of document: review, article, article in press, conference paper, short survey, chapter, erratum, letter.

Search filter: Language of publication: English, Spanish.

Studies retrieved = 220

PsycINFO (date 12/02/2024)

tiab((MAINSUBJECT.EXACT.EXPLODE "Neoplasms" OR (oncolo* OR cancer* OR metasta*))) AND tiab(suicide OR suicides OR suicidal OR parasuicide OR parasuicides OR fatal-attempt OR fatal-attempts OR euthanasia OR assisted-death OR assisted-deaths OR hasten-death OR hastened-death) AND title(review OR meta-analysis OR meta-review OR meta-analytic-review OR metaanalysis OR meta-analyses OR overview* OR umbrella)

Search filter: Type of document: scholarly publications.

Search filter: Language of publication: English, Spanish.

Studies retrieved = 58

PubMed (date 12/02/2024)

(neoplasms [mh] OR oncolo* [tiab] OR cancer* [tiab] OR metasta* [tiab]) AND (suicide [tiab] OR suicides [tiab] OR suicidal [tiab] OR parasuicide [tiab] OR parasuicides [tiab] OR fatal-attempt [tiab] OR fatal-attempts [tiab] OR euthanasia [tiab] OR assisted-death [tiab] OR assisted-deaths [tiab] OR hasten-death [tiab] OR hastened-death [tiab]) AND (review [title] OR meta-analysis [title] OR meta-review [title] OR meta-analytic-review [title] OR metaanalysis [title] OR meta-analyses [title] OR overview* [title] OR umbrella [title])

Studies retrieved = 205

Search filter: Language of publication: English, Spanish.
