# Supplementary material for: Suicidal Ideation, Suicide Attempts, and Suicide Mortality in Cancer: An Overview of Systematic Reviews with Meta-Analysis
Source: Cancers (Basel). 2025 May 27;17(11):1788. doi: 10.3390/cancers17111788 (PMC12153619; doi:10.3390/cancers17111788)
Supplement: Supplementary file 1 [file cancers-17-01788-s001.zip › Suppl File 2 Characteristics of SRs.pdf]

Supplementary file 2. Systematic reviews: characteristics.

Study and year of publication	Statistical Analysis	The certainty of evidence (GRADE) or Methodological quality assessment of primary studies	Population	Outcomes	Studies (K) and participants (N)		Main findings
Amiri and Behnezhad 2020	Meta-regression: No<br><br>Subgroup meta-analysis: Yes<br><br>Sensitivity analysis: No	GRADE: No<br><br>Methodological quality assessment: Effective Public Health Practice Project Criteria.	Different cancer sites	Standardized mortality by suicide ratio (SMR)	k=22 (overall)	N=14,502,575 (overall)<br>(Data from 20 studies. Two studies did not report data)	Overall: SMR (95%CI)= 1.55 (1.37–1.74), I^2= 96.9%<br>High risk of suicide mortality<br><br>Subgroup meta-analysis by geographical region<br>Asia: SMR (95%CI)= 1.18 (0.94–1.49), I^2= 78.4%, k=4<br>Europe: SMR (95%CI)= 1.52 (1.36–1.70), I^2= 93.2%, k=14<br>Americas (North America): SMR (95%CI)= 1.54 (1.37–1.75), I^2= 83.2%, k=3<br><br>Subgroup meta-analysis by cancer site<br>Esophagus, stomach, pancreas, liver: SMR (95%CI)= 2.06 (1.32–3.23), I^2= 91.2%, k=8<br>Colon and rectum: SMR (95%CI)= 1.57 (1.26–1.97), I^2= 87.5%, k=7<br>Bronchus, trachea, lung: SMR (95%CI)= 3.07 (2.20–4.28), I^2= 95.9%, k=8<br>Breast: SMR (95%CI)= 1.24 (1.03–1.48), I^2= 87.8%, k=8<br>Female genital organs: SMR (95%CI)= 1.26 (0.79–1.99), I^2= 94.3%, k=6<br>Prostate: SMR (95%CI)= 1.71 (1.38–2.12), I^2= 88.9%, k=7<br>Melanoma and skin: SMR (95%CI)= 0.93 (0.75–1.16), I^2= 87.8%, k=4<br><br>*The meta-analyses by subgroup of esophagus…, bronchus…,and melanoma/skin were not included in the main text since they included different body part that another review separately evaluated.
Brunckhorst et al. 2021		GRADE: No	Prostate cancer	Prevalence suicidal ideation	Suicide ideation: k= 8 (overall)	Suicide ideation: N=6,173 (overall)	Suicidal ideation: 9.85% (95%CI 7.31-12.70%), I^2= 88.17%<br><br>Subgroup meta-analysis by geographical region

	Meta-regression: No<br><br>Subgroup meta-analysis: Yes<br><br>Sensitivity analysis: Yes	Methodological quality assessment: The Hoy Risk of Bias Tool.		Crude suicide mortality rate per 100,000 person years	Suicide mortality: k=12 (overall)	Suicide mortality: N=13,854,154 (overall)	Europa: 6.73% (95%CI 4.53-9.31), I^2= UR, k=3<br>North America: 12.25% (95%CI 10.70-13.88), I^2= 0%, k=4<br><br>Suicide mortality: 47.1 per 100,000 person years (95%CI 39.85-54.96), I^2= 96.76%<br><br>Subgroup meta-analysis by geographical region<br>Europa: 54.38 per 100,000 person years (95%CI 41.15-69.44), I^2= 94.57%, k=6<br>North America: 43.95 per 100,000 person years (95%CI 33.84-55.34), I^2= 97.95%, k=5
Ding et al. 2023	Meta-regression: Yes<br>Subgroup meta-analysis: No subgroups for suicidal ideation<br>Sensitivity analysis: Yes	GRADE: No<br>Methodological quality assessment: The Agency for Healthcare Research and Quality checklist.	Different cancer sites (no subgroup meta-analysis by cancer site was conducted for the outcome of interest)	Prevalence suicidal ideation	k=12 (overall)	UR	Overall: 24.95% (95%CI 10.96–38.95%), I^2 = 99.43%<br>(all studies came from China)
Du et al. 2020<br><br>REFERNCES FROM CANCER SITES WERE NOT SPECIFIED	Meta-regression: Yes<br>Subgroup meta-analysis: Yes<br>Sensitivity analysis: No	GRADE: No<br>Methodological quality assessment: The Newcastle Ottawa Scale.	Different cancer sites	Incidence suicide death per 100,000 person years	k=36 (overall)	The total sample could not be calculated since some studies did not report the number of participants	Overall: 39.72 per 100,000 person-years (95%CI, 33.91–46.52), I^2 = 99.6%<br><br>Subgroup by geographical region<br>Asia: 61.02 per 100,000 person-years (95%CI, 53.66–69.40), k=6<br>Europe: 39.28 per 100,000 person-years (95%CI, 26.95–57.25), k=16<br>(Schairer et al. 2006 appears twice)<br>North America: 32.27 per 100,000 person-years (95%CI,28.19–36.94), k=15<br>Oceania: 24.07 per 100,000 person-years (95%CI, 20.78–27.88), k=2<br><br>Subgroup meta-analysis by cancer site<br>Sarcoma: 60.99 per 100,000 person-years (95%CI, 17.37–214.19), k=2<br>Breast: 11.17 per 100,000 person-years (95%CI, 7.15–17.45), k=5<br>Central Nervous System (CNS): 27.67 per 100,000 person-years (95%CI, 18.49–41.38), k=5

							Colorectum: 24.55 per 100,000 person-years (95%CI, 18.89–31.91), k=6<br>Gastric: 51.83 per 100,000 person-years (95%CI, 36.18–74.26), k=6<br>Esophagus: 87.71 per 100,000 person-years (95%CI, 27.42–280.54), k=3<br>Liver: 38.13 per 100,000 person-years (95%CI, 15.64–92.96), k=3<br>Pancreas: 75.39 per 100,000 person-years (95%CI, 41.80–135.97), k=4<br>Prostate: 35.82 per 100,000 person-years (95%CI, 26.29–48.80), k=9<br>Testis: 32.01 per 100,000 person-years (95%CI, 18.58–55.15), k=6<br>Cervix: 8.83 per 100,000 person-years (95%CI, 6.38–12.23), k=4<br>Ovary: 12.57 per 100,000 person-years (95%CI, 6.83–23.11), k=3<br>Uterus: 6.19 per 100,000 person-years (95%CI, 4.41–8.70), k=3<br>Vulvar: 7.40 per 100,000 person-years (95%CI, 2.78–19.72), k=2<br>Head and neck: 53.76 per 100,000 person-years (95%CI, 19.92–145.10), k=3<br>Non-Hodgkin Lymphoma: 21.38 per 100,000 person-years (95%CI, 8.08–56.62), k=2<br>Leukemias: 17.91 per 100,000 person-years (95%CI, 6.44–49.81), k=2<br>Hodgkin Lymphoma: 24.93 per 100,000 person-years (95%CI, 9.40–66.12), k=2<br>Lung: 44.89 per 100,000 person-years (95%CI, 26.37–76.43), k=6<br>Bladder: 26.90 per 100,000 person-years (95%CI, 16.81–43.04), k=4<br>Kidney: 30.61 per 100,000 person-years (95%CI, 22.14–42.34), k=4
Guo et al. 2018	Meta-regression: Yes<br>Subgroup meta-analysis: Yes<br>Sensitivity analysis: Yes	GRADE: No<br>Methodological quality assessment: The Newcastle Ottawa Scale	Prostate cancer	Risk of suicidal ideation	k=8 (overall)	N=2,123,687 (overall)	An overall increased RR of suicide of 2.01 (95%CI: 1.52–2.64), I^2= 91.8% among people with prostate cancer in comparison to people free prostate cancer during the first 12 months after diagnosis<br><br>Subgroup meta-analysis by geographical region<br>Europe (only in Sweden): RR 1.80 (95%CI 1.27-2.57), I^2 = 92%, k=3<br><br>*The United States and Canada were separately analyzed.
Guo et al. 2021	Meta-regression: Yes	GRADE: No<br>Methodological quality assessment:	Bladder cancer	Risk of suicidal ideation	k=5 (overall)	N=563,680 (overall)	Higher risk of suicide among people with bladder cancer compared with people without bladder cancer HR 1.90 (95%CI 1.29−2.81), I^2= 81.2%

	Subgroup meta-analysis: Yes<br>Sensitivity analysis: Yes	The Newcastle Ottawa Scale						Subgroup meta-analysis by geographical region<br>*The United States and Canada were separately analyzed.
Heinrich et al. 2022<br><br>REFERNCES FROM CANCER SITES WERE NOT SPECIFIED	Meta-regression: Yes<br>Subgroup meta-analysis: Yes<br>Sensitivity analysis: Yes	GRADE: No<br><br>Methodological quality assessment: The Newcastle Ottawa Scale.	Different cancer sites	Risk of mortality by suicide	k=28 (overall)	N=22,407,330 (overall)		SMR 1.85 (95%CI 1.55-2.20), I^2 = 99.37%<br>An 85% increased suicide mortality rate among patients with cancer compared with the general population.<br><br>Subgroup meta-analysis by geographical region<br>Americas (only the USA): SMR 2.77 (95%CI 1.62-4.74), I^2 = 99.37%, k=UR<br>Europe: SMR 1.51 (95%CI 1.33-1.72), I^2 = 99.37%, k=UR<br>Asia: SMR 1.97 (95%CI 1.44-2.70), I^2 = 99.37%, k=UR<br>Oceania (only Australia): SMR 1.63 (95%CI 1.40-1.90), I^2 = 99.37%, k=UR<br><br>Subgroup meta-analysis by cancer site<br>Endometrium: SMR 1.21 (95%CI, 0.68–2.16), k=3<br>Skin: SMR 1.32 (95%CI, 0.72–2.43), k=6<br>Female genital system: SMR 1.35 (95%CI, 0.59–3.07), k=5<br>Melanoma: SMR 1.38 (95%CI, 0.35–5.51), k=3<br>Bladder: SMR 1.39 (95%CI, 1.09–1.78), k=4<br>Thyroid: SMR 1.39 (95%CI, 0.42–4.55), k=4<br>Prostate: SMR 1.46 (95%CI, 1.20–1.77), k=11<br>Breast: SMR 1.49 (95%CI, 0.94–2.36), k=10<br>Urinary system: SMR 1.79 (95%CI, 0.96–3.34), k=3<br>Colorectum: SMR 1.80 (95%CI, 1.44–2.25), k=10<br>Testicular: SMR 1.89 (95%CI, 0.64–5.58), k=5<br>Hematological system: SMR 1.94 (95%CI, 1.25–3.00), k=6<br>Leukemia: SMR 2.09 (95%CI, 0.83–5.24), k=3<br>Kidney: SMR 2.18 (95%CI, 0.74–6.43), k=3<br>Non-Hodgkin Lymphoma: SMR 2.39 (95%CI, 0.84–6.81), k=3<br>Stomach: SMR 2.42 (95%CI, 1.40–4.19), k=7<br>Penis: SMR 2.58 (95%CI, 0.90–7.42), k=2<br>Head and neck: SMR 2.87 (95%CI, 2.00–4.12), k=8<br>Cervix: SMR 2.99 (95%CI, 0.54–16.43), k=3<br>Lung: SMR 3.14 (95%CI, 2.38–4.15), k=12<br>Central nervous system: SMR 3.15 (95%CI, 1.31–7.60), k=6<br>Liver: SMR 3.44 (95%CI, 1.48–7.98), k=4

							Ovary: SMR 4.08 (95%CI, 0.82–20.30), k=3<br>Hodgkin Lymphoma: SMR 4.24 (95%CI, 0.23–79.03), k=2<br>Biliary system: SMR 4.30 (95%CI, 0.76–24.41), k=2<br>Esophagus: SMR 6.01 (95%CI, 2.14–16.86), k=4<br>Pancreas: SMR 6.42 (95%CI, 1.60–25.76), k=4<br>Bone and cartilage: SMR 9.59 (95%CI, 1.54–59.77), k=2<br>Mesothelioma: SMR 13.07 (95%CI, 1.61–105.80), k=2
Hofmann et al. 2023	Meta-regression: Yes<br>Subgroup meta-analysis: Yes<br>Sensitivity analysis: No	GRADE: No<br><br>Methodological quality assessment: The Newcastle Ottawa Scale.	Lung cancer	Risk of mortality by suicide	k=12 (overall without overlapping studies)	Some studies did not report the number of people with lung cancer	SMR 2.95 (95%CI 2.42–3.60), I^2=90.46%<br>The suicide mortality risk was increased 2.95-fold compared to the general population<br><br>Subgroup meta-analysis by geographical region<br>Americas (only the United States): SMR 5.13 (95%CI 3.71-7.10), k=7<br>Europe: SMR 2.90 (95%CI 2.39-3.52), k=11<br>Asia: SMR 3.21 (95%CI 2.65-3.89), I^2 = 99.37%, k=4
Lee et al. 2023	Meta-regression: Yes<br>Subgroup meta-analysis: Yes<br>Sensitivity analysis: Yes	GRADE: No<br><br>Methodological quality assessment: The Joanna Briggs Institute critical appraisal checklist.	Different cancer sites	Risk of mortality by suicide per 100,000 Person-Years	k=3 (overall)	UR	RR 1.63 (95%CI 0.78-3.41), I^2= 50%<br>The results did not reach statistical significance
Qin et al. 2022	Meta-regression: No<br>Subgroup meta-analysis: Yes<br>Sensitivity analysis: Yes	GRADE: No<br><br>Methodological quality assessment: The Newcastle Ottawa Scale.	Different cancer sites	Risk of mortality by suicide<br><br>Prevalence of suicide	Suicide mortality risk k=3 (overall)<br>Prevalence: k=5 (overall)	Suicide mortality risk: UR<br>Prevalence: N=43,396 (overall)	RR 2.64 (95%CI 2.26-3.10), I^2= 29.9%<br><br>Prevalence: 8.02% (95%CI 0.70-21.90), I^2= 97.2%
Rafiei et al. 2023	Meta-regression: Yes	GRADE: No	Different cancer sites	Prevalence suicide ideation	k=UR	Suicide ideation: 4,030 out of 67,169 patients	Suicide ideation: 6%<br>Suicide attempt: 60%<br>Suicide death: 34%

	Subgroup meta-analysis: Yes<br>Sensitivity analysis: No	Methodological quality assessment: The Newcastle Ottawa Scale.		Prevalence suicide attempt<br>Prevalence suicide death		Suicide attempt: 40,300 out of 67,169 patients<br><br>Suicide death: 22,839 out of 67,169 patients	*Subgroup meta-analyses by geographical region or cancer site were not independently conducted by type of suicide
Ravaioli et al. 2020	Meta-regression: No<br>Subgroup meta-analysis: Yes<br>Sensitivity analysis: Yes	GRADE: No<br><br>Methodological quality assessment: UR	UR	Risk of mortality by suicide	k=14 (overall)	N=UR	SMR 1.7 (95%CI 1.5-1.9), I^2= 98.4%<br>There was an increased of mortality by suicide

Note: HR: Hazard ratio; RR: Relative risk; SMR: Standardized Mortality Ratio, UR: unreported.
