# Supplementary material for: Suicidal Ideation, Suicide Attempts, and Suicide Mortality in Cancer: An Overview of Systematic Reviews with Meta-Analysis
Source: Cancers (Basel). 2025 May 27;17(11):1788. doi: 10.3390/cancers17111788 (PMC12153619; doi:10.3390/cancers17111788)
Supplement: Supplementary file 1 [file cancers-17-01788-s001.zip › Suppl File 4 Excluded studies list.pdf]

Supplementary file 4. Excluded studies after analyzing the full text: list (n=75).

Cancer was combined with other populations (n= 1)

Conference abstract (n=1)

Impossibility to get access to the full text (n= 2)

No meta-analysis (n= 57)

No objective of this study (n= 7)

Overview (n= 4)

Protocol (n= 3)

*Note: Manual searches appear after this table.

	**Excluded reviews**	**Reason**
1.	A review of successful hospital suicides and their aftermaths. Hospital Security and Safety Management, 01 Apr 1991, 11(12):5-10 PMID: 10112261.	Impossibility to get access to the full text.<br><br>Note: The abstract does not report any information that leads us to suspect that this review was a systematic review with meta-analysis.
2.	Abdel-Bakky MS, Amin E, Faris TM, Abdellatif AAH. Mental depression: Relation to different disease status, newer treatments and its association with COVID-19 pandemic (Review). Mol Med Rep. 2021 Dec;24(6):839. doi: 10.3892/mmr.2021.12479.	No meta-analysis.
3.	Al-Azri M, Al-Awisi H, Al-Moundhri M. Coping with a diagnosis of breast cancer-literature review and implications for developing countries. Breast J. 2009 Nov-Dec;15(6):615-22. doi: 10.1111/j.1524-4741.2009.00812.x.	No meta-analysis.
4.	Anguiano L, Mayer DK, Piven ML, Rosenstein D. A literature review of suicide in cancer patients. Cancer Nurs. 2012 Jul-Aug;35(4):E14-26. doi: 10.1097/NCC.0b013e31822fc76c.	No meta-analysis.
5.	Balk EM, Earley A, Avendano EA, Raman G. Long-Term Health Outcomes in Women With Silicone Gel Breast Implants: A Systematic Review. Ann Intern Med. 2016 Feb 2;164(3):164-75. doi: 10.7326/M15-1169.	Cancer was combined with other populations.<br><br>Note: Studies evaluating cancer were pooled with studies evaluating other populations.
6.	Beardsley C, Brown K, Sandroussi C. Euthanasia and surgeons: an overview of the Victorian Voluntary Assisted Dying Act 2017 and its relevance to surgical practice in Australia. ANZ J Surg. 2018 Oct;88(10):956-958. doi: 10.1111/ans.14513.	No meta-analysis.
7.	Belcher SM, Hausmann EA, Cohen SM, Donovan HS, Schlenk EA. Examining the relationship between multiple primary cancers and psychological distress: A review of current literature. Psychooncology. 2017 Dec;26(12):2030-2039. doi: 10.1002/pon.4299.	No meta-analysis.
8.	Bleyer A, Barr R. Cancer in young adults 20 to 39 years of age: overview. Semin Oncol. 2009 Jun;36(3):194-206. doi: 10.1053/j.seminoncol.2009.03.003.	No meta-analysis.
9.	Borges do Nascimento IJ, Marcolino MS, Abdulazeem HM, Weerasekara I, Azzopardi-Muscat N, Gonçalves MA, Novillo-Ortiz D. Impact of Big Data Analytics on People's Health: Overview of	Overview.

	Systematic Reviews and Recommendations for Future Studies. J Med Internet Res. 2021 Apr 13;23(4):e27275. doi: 10.2196/27275.	
10.	Bottomley A. Depression in cancer patients: a literature review. Eur J Cancer Care (Engl). 1998 Sep;7(3):181-91. doi: 10.1046/j.1365-2354.1998.00100.x.	No meta-analysis.
11.	Calabria B, Degenhardt L, Hall W, Lynskey M. Does cannabis use increase the risk of death? Systematic review of epidemiological evidence on adverse effects of cannabis use. Drug Alcohol Rev. 2010 May;29(3):318-30. doi: 10.1111/j.1465-3362.2009.00149.x.	No meta-analysis.
12.	Calati R, Fang F, Mostofsky E, Shen Q, Di Mattei VE, Garcia-Foncillas J, Baca-Garcia E, Cipriani A, Courtet P. Cancer and suicidal ideation and behaviours: protocol for a systematic review and meta-analysis. BMJ Open. 2018 Aug 10;8(8):e020463. doi: 10.1136/bmjopen-2017-020463.	Protocol.
13.	Calati R, Filipponi C, Mansi W, Casu D, Peviani G, Gentile G, Tambuzzi S, Zoja R, Fornaro M, Lopez-Castroman J, Madeddu F. Cancer diagnosis and suicide outcomes: Umbrella review and methodological considerations. J Affect Disord. 2021 Dec 1;295:1201-1214. doi: 10.1016/j.jad.2021.08.131.	Overview.
14.	Campeny E, López-Pelayo H, Nutt D, Blithikioti C, Oliveras C, Nuño L, Maldonado R, Florez G, Arias F, Fernández-Artamendi S, Villalbí JR, Sellarès J, Ballbè M, Rehm J, Balcells-Olivero MM, Gual A. The blind men and the elephant: Systematic review of systematic reviews of cannabis use related health harms. Eur Neuropsychopharmacol. 2020 Apr;33:1-35. doi: 10.1016/j.euroneuro.2020.02.003.	Overview.
15.	Carreira H, Williams R, Müller M, Harewood R, Stanway S, Bhaskaran K. Associations Between Breast Cancer Survivorship and Adverse Mental Health Outcomes: A Systematic Review. J Natl Cancer Inst. 2018 Dec 1;110(12):1311-1327. doi: 10.1093/jnci/djy177. Erratum in: J Natl Cancer Inst. 2020 Jan 1;112(1):118.	No meta-analysis.
16.	Caruso R, Nanni MG, Riba M, Sabato S, Mitchell AJ, Croce E, Grassi L. Depressive spectrum disorders in cancer: prevalence, risk factors and screening for depression: a critical review. Acta Oncol. 2017 Feb;56(2):146-155. doi: 10.1080/0284186X.2016.1266090.	No objective of this study.
17.	Cassem EH. Depressive disorders in the medically ill. An overview. Psychosomatics. 1995 Mar-Apr;36(2):S2-10. doi: 10.1016/S0033-3182(95)71698-X.	No meta-analysis.
18.	Chan ASW, Leung LM, Wong FKC, Ho JMC, Tam HL, Tang PMK, Yan E. Needs and experiences of cancer care in patients' perspectives among the lesbian, gay, bisexual, transgender and queer community: a systematic review. Soc Work Health Care. 2023 Jul-Dec;62(8-9):263-279. doi: 10.1080/00981389.2023.2226182.	No meta-analysis.
19.	Chen J, Ping Z, Hu D, Wang J, Liu Y. Risk factors associated with suicidal ideation among cancer patients: a systematic review and meta-analysis. Front Psychol. 2024 Jan 8;14:1287290. doi: 10.3389/fpsyg.2023.1287290.	No objective of this study.
20.	Costanza A, Zenga F, Rudà R, Amerio A, Aguglia A, Serafini G, Amore M, Bondolfi G, Berardelli I, Nguyen KD. Suicidality in Patients with Brain Tumors: A Brief Literature Review with Clinical Exemplar. Medicina (Kaunas). 2020 Dec 21;56(12):725. doi: 10.3390/medicina56120725.	No meta-analysis.
21.	Cotter AR, Vuong K, Mustelin L, Yang Y, Rakhmankulova M, Barclay CJ, Harris RP. Do psychological harms result from being	No meta-analysis.

	labelled with an unexpected diagnosis of abdominal aortic aneurysm or prostate cancer through screening? A systematic review. BMJ Open. 2017 Dec 12;7(12):e017565. doi: 10.1136/bmjopen-2017-017565. Erratum in: BMJ Open. 2018 Jan 21;8(1):e017565corr1.	
22.	de Graeff A, Dean M. Palliative sedation therapy in the last weeks of life: a literature review and recommendations for standards. J Palliat Med. 2007 Feb;10(1):67-85. doi: 10.1089/jpm.2006.0139.	No meta-analysis.
23.	Dines P, Hu W, Sajatovic M. Depression in Later-life: An Overview of Assessment and Management. Psychiatr Danub. 2014 Nov;26 Suppl 1:78-84.	No meta-analysis.
24.	Du L, Shi HY, Qian Y, Jin XH, Li Y, Yu HR, Liu XM, Fu XL, Chen HL. Association between social support and suicidal ideation in patients with cancer: A systematic review and meta-analysis. Eur J Cancer Care (Engl). 2021 Mar;30(2):e13382. doi: 10.1111/ecc.13382.	No objective of this study.
25.	Eastman P, Le B. Palliative care after attempted suicide in the absence of premorbid terminal disease: a case series and review of the literature. J Pain Symptom Manage. 2013 Feb;45(2):305-9. doi: 10.1016/j.jpainsymman.2012.01.008.	No meta-analysis.
26.	Favril L, Yu R, Geddes JR, Fazel S. Individual-level risk factors for suicide mortality in the general population: an umbrella review. Lancet Public Health. 2023 Nov;8(11):e868-e877. doi: 10.1016/S2468-2667(23)00207-4.	Overview.
27.	Fervaha G, Izard JP, Tripp DA, Rajan S, Leong DP, Siemens DR. Depression and prostate cancer: A focused review for the clinician. Urol Oncol. 2019 Apr;37(4):282-288. doi: 10.1016/j.urolonc.2018.12.020.	No meta-analysis.
28.	Fiske A, O'Riley AA, Widoe RK. Physical Health and Suicide in Late Life: An Evaluative Review, Clinical Gerontologist. 2008;31: 31-50. doi: 10.1080/07317110801947151	No meta-analysis.
29.	Friedlander AH, Rosenbluth SC, Rubin RT. The adult suicide-prone patient: a review of the medical literature and implications for oral and maxillofacial surgeons. J Oral Maxillofac Surg. 2012 May;70(5):1253-60. doi: 10.1016/j.joms.2011.02.024.	No meta-analysis.
30.	Gan LL, Gong S, Kissane DW. Mental state of demoralisation across diverse clinical settings: A systematic review, meta-analysis and proposal for its use as a 'specifier' in mental illness. Aust N Z J Psychiatry. 2022 Sep;56(9):1104-1129. doi: 10.1177/00048674211060746.	No meta-analysis.<br>Note: No meta-analysis regarding the association of interest.
31.	Graf J, Stengel A. Psychological Burden and Psycho-Oncological Interventions for Patients With Hepatobiliary Cancers-A Systematic Review. Front Psychol. 2021 May 5;12:662777. doi: 10.3389/fpsyg.2021.662777.	No meta-analysis.
32.	Grummitt LR, Kreski NT, Kim SG, Platt J, Keyes KM, McLaughlin KA. Association of Childhood Adversity With Morbidity and Mortality in US Adults: A Systematic Review. JAMA Pediatr. 2021 Dec 1;175(12):1269-1278. doi: 10.1001/jamapediatrics.2021.2320.	No objective of this study.
33.	Henry OA, Sheedy MT, Beischer NA. When is a maternal death a maternal death? A review of maternal deaths at the Mercy Maternity Hospital, Melbourne. Med J Aust. 1989 Dec 4-18;151(11-12):628-31. doi: 10.5694/j.1326-5377.1989.tb139635.x.	No meta-analysis.
34.	Hicks MH. Physician-assisted suicide: a review of the literature concerning practical and clinical implications for UK doctors. BMC Fam Pract. 2006 Jun 22;7:39. doi: 10.1186/1471-2296-7-39.	No meta-analysis.

35.	Hong YT, Lin YA, Pan YX, Lin JL, Lin XJ, Zhang J, Huang FF. Understanding factors influencing demoralization among cancer patients based on the bio-psycho-social model: A systematic review. Psychooncology. 2022 Dec;31(12):2036-2049. doi: 10.1002/pon.6023.	No meta-analysis.
36.	Honkaniemi H, Bacchus-Hertzman J, Fritzell J, Rostila M. Mortality by country of birth in the Nordic countries - a systematic review of the literature. BMC Public Health. 2017 May 25;17(1):511. doi: 10.1186/s12889-017-4447-9.	No meta-analysis.
37.	Iannucci J, Nierenberg B. Suicide and suicidality in children and adolescents with chronic illness: A systematic review. Aggression and Violent Behavior. 2022;64: 101581.	No meta-analysis.
38.	Inoue, K., Tanii, H., Kaiya, H., Okazaki, Y., & Fukunaga, T. (2008). The relationship between suicide and physical illness in Japan: A review. International Medical Journal, 15(1), 35–37.	Impossibility to get access to the full text.<br>Note: The abstract does not report any information that leads us to suspect that this review was a systematic review with meta-analysis.
39.	Jaiswal R, Alici Y, Breitbart W. A comprehensive review of palliative care in patients with cancer. Int Rev Psychiatry. 2014 Feb;26(1):87-101. doi: 10.3109/09540261.2013.868788.	No meta-analysis.
40.	Kazlauskiene J, Navickas A, Lesinskienė S, Bulotiene G. Risk factors for suicide in cancer patients and preventive measures: a literature review Subtitle: Cancer patient's suicides and prevention. Arch Psych Psych 2022;24(4):68-77.	No meta-analysis.
41.	Kim H, Kim K, Kim YH. Associations between mental illness and cancer: a systematic review and meta-analysis of observational studies. Eur Rev Med Pharmacol Sci. 2022 Jul;26(14):4997-5007. doi: 10.26355/eurrev_202207_29286.	No objective of this study.
42.	Kolva E, Hoffecker L, Cox-Martin E. Suicidal ideation in patients with cancer: A systematic review of prevalence, risk factors, intervention and assessment. Palliat Support Care. 2020 Apr;18(2):206-219. doi: 10.1017/S1478951519000610.	No meta-analysis.
43.	Li C, Zhang M, Wang Q, Jiang K, Ye Y. Risk of suicide in patients with thyroid cancer: protocol for a systematic review and meta-analysis. BMJ Open. 2024 Jan 24;14(1):e080210. doi: 10.1136/bmjopen-2023-080210.	Protocol.
44.	Lin CC, Her YN. Demoralization in cancer survivors: an updated systematic review and meta-analysis for quantitative studies. Psychogeriatrics. 2024 Jan;24(1):35-45. doi: 10.1111/psyg.13037.	No meta-analysis.<br>Note: No meta-analysis of interest.
45.	Liu W, Liu L, Liu A, Jia S, Liu T, Boldt G, Qu M, Ball I. Incidence and risk factors for euthanasia or physician-assisted suicide in oncology patients: A systematic review. Radiotherapy and Oncology. 2021;163(Suppl 1):S28. https://doi.org/10.1016/S0167-8140(21)08937-4.	Conference abstract.
46.	Liu B, Lee K, Sun C, Wu D, Lim PY. Systematic review on factors associated with self-perceived burden among cancer patients. Support Care Cancer. 2022 Oct;30(10):8417-8428. doi: 10.1007/s00520-022-07129-9.	No meta-analysis.
47.	Lupton A, Abu-Suwa H, Bolton GC, Golden C. The implications of brain tumors on aggressive behavior and suicidality: A review. Aggresion and Violent Behavior. 2020;54: 101416.	No meta-analysis.
48.	Madsen R, Uhrenfeldt L. Palliative patients' and their significant others' experiences of transitions concerning organizational, psychosocial and existential issues during the course of incurable	Protocol.

	cancer: a systematic review protocol. JBI Database of Systematic Reviews and Implementation Reports. 2014;12:9-25. doi: 10.11124/jbisrir-2014-1161.	
49.	Maria Alexandra Stanescu A, Totan A, Mircescu D, Diaconescu S, Gabriel Bratu O, Fekete L, László Fekete G, Boda D, Cristina Diaconu C. Assessment of suicidal behavior in dermatology (Review). Exp Ther Med. 2020 Jul;20(1):73-77. doi: 10.3892/etm.2019.8145.	No meta-analysis.
50.	Massa E, Donisi C, Liscia N, Madeddu C, Impera V, Mariani S, Scartozzi M, Lai E. The Difficult Task of Diagnosing Depression in Elderly People with Cancer: A Systematic Review. Clin Pract Epidemiol Ment Health. 2021 Dec 31;17(1):295-306. doi: 10.2174/1745017902117010295.	No meta-analysis.
51.	McDonough J, Eliott J, Neuhaus S, Reid J, Butow P. Health-related quality of life, psychosocial functioning, and unmet health needs in patients with sarcoma: A systematic review. Psychooncology. 2019 Apr;28(4):653-664. doi: 10.1002/pon.5007.	No meta-analysis.
52.	Mendes MVC, Santos SLD, Ceballos AGDC, Furtado BMASM, Bonfim CVD. Risk factors for suicide in individuals with cancer: an integrative literature review. Rev Bras Enferm. 2021 May 21;74(suppl 3):e20190889. English, Portuguese. doi: 10.1590/0034-7167-2019-0889.	No meta-analysis.
53.	Mercadante S, Porzio G, Valle A, Fusco F, Aielli F, Costanzo V; Home Care Italy Group. Palliative sedation in patients with advanced cancer followed at home: a systematic review. J Pain Symptom Manage. 2011 Apr;41(4):754-60. doi: 10.1016/j.jpainsymman.2010.07.013.	No meta-analysis.
54.	Mofatteh M, Mashayekhi MS, Arfaie S, Chen Y, Malhotra AK, Alvi MA, Sader N, Antonick V, Fatehi Hassanabad M, Mansouri A, Das S, Liao X, McIntyre RS, Del Maestro R, Turecki G, Cohen-Gadol AA, Zadeh G, Ashkan K. Suicidal ideation and attempts in brain tumor patients and survivors: A systematic review. Neurooncol Adv. 2023 May 12;5(1):vdad058. doi: 10.1093/noajnl/vdad058.	No meta-analysis.
55.	Oliveri S, Ferrari F, Manfrinati A, Pravettoni G. A Systematic Review of the Psychological Implications of Genetic Testing: A Comparative Analysis Among Cardiovascular, Neurodegenerative and Cancer Diseases. Front Genet. 2018 Dec 10;9:624. doi: 10.3389/fgene.2018.00624.	No meta-analysis.
56.	Parpa E, Tsilika E, Gennimata V, Mystakidou K. Elderly cancer patients' psychopathology: a systematic review: aging and mental health. Arch Gerontol Geriatr. 2015 Jan-Feb;60(1):9-15. doi: 10.1016/j.archger.2014.09.008.	No meta-analysis.
57.	Pascale A, Beal MW, Fitzgerald T. Rethinking the Well Woman Visit: A Scoping Review to Identify Eight Priority Areas for Well Woman Care in the Era of the Affordable Care Act. Womens Health Issues. 2016 Mar-Apr;26(2):135-46. doi: 10.1016/j.whi.2015.11.003.	No meta-analysis.
58.	Pedersen C, Tariman JD. Beliefs and Attitudes of American Nurses on Physician Assisted Suicide: An Integrative Literature Review. Journal of nursing practice applications & reviews of research. 2018; 8:24-38. https://doi.org/10.13178/jnparr.2018.0801.0805	No meta-analysis.
59.	Pham H, Torres H, Sharma P. Mental health implications in bladder cancer patients: A review. Urol Oncol. 2019 Feb;37(2):97-107. doi: 10.1016/j.urolonc.2018.12.006.	No meta-analysis.
60.	Robinson S, Kissane DW, Brooker J, Burney S. A systematic review of the demoralization syndrome in individuals with	No meta-analysis.

	progressive disease and cancer: a decade of research. J Pain Symptom Manage. 2015 Mar;49(3):595-610. doi: 10.1016/j.jpainsymman.2014.07.008.	
61.	Robson A, Scrutton F, Wilkinson L, MacLeod F. The risk of suicide in cancer patients: a review of the literature. Psychooncology. 2010 Dec;19(12):1250-8. doi: 10.1002/pon.1717.	No meta-analysis.
62.	Sanmarchi F, Esposito F, Adorno E, De Dominicis F, Fantini MP, Golinelli D. The impact of the SARS-CoV-2 pandemic on cause-specific mortality patterns: a systematic literature review. Z Gesundh Wiss. 2022 Sep 26:1-19. doi: 10.1007/s10389-022-01755-7.	No meta-analysis.
63.	Sherk C, Thomas H, Wilson DM, Evans CE. Health consequences of selected lifestyle factors: a review of the evidence, part 2. Can Fam Physician. 1985 Jan;31:129-39.	No meta-analysis.
64.	Simpson WG, Klaassen Z, Jen RP, Hughes WM 5th, Neal DE Jr, Terris MK. Analysis of Suicide Risk in Patients with Penile Cancer and Review of the Literature. Clin Genitourin Cancer. 2018 Apr;16(2):e257-e261. doi: 10.1016/j.clgc.2017.09.011.	No meta-analysis.
65.	Smith JD, Shuman AG, Riba MB. Psychosocial Issues in Patients with Head and Neck Cancer: an Updated Review with a Focus on Clinical Interventions. Curr Psychiatry Rep. 2017 Sep;19(9):56. doi: 10.1007/s11920-017-0811-9.	No meta-analysis.
66.	Spillane A, Larkin C, Corcoran P, Matvienko-Sikar K, Riordan F, Arensman E. Physical and psychosomatic health outcomes in people bereaved by suicide compared to people bereaved by other modes of death: a systematic review. BMC Public Health. 2017 Dec 12;17(1):939. doi: 10.1186/s12889-017-4930-3.	No meta-analysis.
67.	Steck N, Egger M, Maessen M, Reisch T, Zwahlen M. Euthanasia and assisted suicide in selected European countries and US states: systematic literature review. Med Care. 2013 Oct;51(10):938-44. doi: 10.1097/MLR.0b013e3182a0f427.	No meta-analysis.
68.	Tang PL, Wang HH, Chou FH. A Systematic Review and Meta-Analysis of Demoralization and Depression in Patients With Cancer. Psychosomatics. 2015 Nov-Dec;56(6):634-43. doi: 10.1016/j.psym.2015.06.005.	No objective of this study.
69.	Tobin MB, Steinberg GD. A review of incidence and relevant risk factors in genitourinary malignancies. Cancer. 2015 Jun 1;121(11):1731-4. doi: 10.1002/cncr.29273.	No meta-analysis.
70.	Treister-Goltzman Y, Peleg R. Fibromyalgia and mortality: a systematic review and meta-analysis. RMD Open. 2023 Jul;9(3):e003005. doi: 10.1136/rmdopen-2023-003005.	No objective of this study.
71.	Twigg JA, Anderson JM, Humphris G, Nixon I, Rogers SN, Kanatas A. Best practice in reducing the suicide risk in head and neck cancer patients: a structured review. Br J Oral Maxillofac Surg. 2020 Nov;58(9):e6-e15. doi: 10.1016/j.bjoms.2020.06.035.	No meta-analysis.
72.	van Deijck RH, Hasselaar JG, Verhagen SC, Vissers KC, Koopmans RT. Determinants of the administration of continuous palliative sedation: a systematic review. J Palliat Med. 2013 Dec;16(12):1624-32. doi: 10.1089/jpm.2013.0173.	No meta-analysis.
73.	Wang Y, Sun H, Ji Q, Wu Q, Wei J, Zhu P. Prevalence, Associated Factors and Adverse Outcomes of Demoralization in Cancer Patients: A Decade of Systematic Review. Am J Hosp Palliat Care. 2023 Nov;40(11):1216-1230. doi: 10.1177/10499091231154887.	No meta-analysis.
74.	Zarogoulidis P, Chatzaki E, Hohenforst-Schmidt W, Goldberg EP, Galaktidou G, Kontakiotis T, Karamanos N, Zarogoulidis K.	No meta-analysis.

	Management of malignant pleural effusion by suicide gene therapy in advanced stage lung cancer: a case series and literature review. Cancer Gene Ther. 2012 Sep;19(9):593-600. doi: 10.1038/cgt.2012.36.	
75.	Zhiqiang L, Bin S, Min F, Yufang L. Leiomyosarcoma of cervical stump following subtotal hysterectomy: a case report and review of literature. Eur J Gynaecol Oncol. 2016;37(1):148-51.	No meta-analysis.
