# Supplementary material for: Suicidal Ideation, Suicide Attempts, and Suicide Mortality in Cancer: An Overview of Systematic Reviews with Meta-Analysis
Source: Cancers (Basel). 2025 May 27;17(11):1788. doi: 10.3390/cancers17111788 (PMC12153619; doi:10.3390/cancers17111788)
Supplement: Supplementary file 1 [file cancers-17-01788-s001.zip › Suppl File 5 Overall Overlap.pdf]

Supplementary file 5. Matrices of evidence and the corrected covered area (CCA) calculations.

Suicide mortality.

$$CCA = \frac{N-r}{rc-r} = \frac{102-59}{413-59} \; \frac{43}{354} = 0.12 = 12\%$$

Note: N is the total number of original studies (including duplicates) in the meta-analyses of interest (the sum of all checked boxes in the citation matrix). Furthermore, r is the number of original studies without accounting for duplicates. Finally, c is the number of systematic reviews included in the evidence matrix (k=7). CCA = corrected covered area.

**Number of studies without accounting for duplicates (r)**	**References of primary research**	**Systematic reviews where appear primary research including primary research duplicates (N)**
1.	Ahn, E., Shin, D.W., Cho, S.I., Park, S., Won, Y.J., Yun, Y.H., 2010. Suicide rates and risk factors among Korean cancer patients, 1993-2005. Cancer Epidemiol. Biomark. Prevent.: Publ. Am. Assoc. Cancer Res. Cosponsored Am. Soc. Prevent. Oncol. 19, 2097–2105.	1. Du et al. 2020<br>2. Hofmann et al. 2023<br>3. Ravaioli et al. 2020
2.	Ahn, M. H., Park, S., Lee, H. B., Ramsey, C. M., Na, R., Kim, S. O., … Hong, J. P. (2015). Suicide in cancer patients within the first year of diagnosis. Psychooncology, 24(5), 601–607. doi: 10.1002/pon.3705	4. Amiri and Behnezhad 2020
3.	Alanee, S., Russo, P., 2012. Suicide in men with testis cancer. Eur. J. Cancer Care 21, 817–821.	5. Du et al. 2020
4.	Allebeck, P.; Bolund, C. Suicides and suicide attempts in cancer patients. Psychol. Med. 1991, 21, 979–984.	6. Hofmann et al. 2023

5.	Anderson, C., Park, E.M., 2018. Suicide rates among patients with cancers of the digestive system. Psychooncology 27, 2274–2280.	7. Du et al. 2020
6.	Barnes JM, Johnson KJ, Grove JL, Srivastava AJ, Osazuwa-Peters N, Perkins SM. Risk of suicide among individuals with a history of childhood cancer. Cancer. 2022;128(3):624-632. doi:10.1002/cncr.33957	8. Lee et al. 2023
7.	Bill-Axelson A, Garmo H, Lambe M, Bratt O, Adolfsson J, Nyberg U, et al. Suicide Risk in Men with Prostate-Specific Antigen-Detected Early Prostate Cancer: A Nationwide Population-Based Cohort Study from PCBaSe Sweden. Eur Urol. 2010;57:390-5.	9. Brunckhorst et al. 2021<br>10. Du et al. 2020
8.	Bjorkenstam, C., Edberg, A., Ayoubi, S., Rosen, M., 2005. Are cancer patients at higher suicide risk than the general population? Scand. J. Publ. Health 33 (3), 208–214.	11. Qin et al. 2022
9.	Bowden, M.B., Walsh, N.J., Jones, A.J., Talukder, A.M., Lawson, A.G., Kruse, E.J., 2017. Demographic and clinical factors associated with suicide in gastric cancer in the United States. J. Gastrointest. Oncol. 8, 897–901.	12. Du et al. 2020
10.	Camidge DR, Stockton DL, Frame S, Wood R, Bain M, Bateman DN. Hospital admissions and deaths relating to deliberate self-harm and accidents within 5 years of a cancer diagnosis: a national study in Scotland, UK. Br J Cancer 2007;96:752e7. https://doi.org/10.1038/sj.bjc.6603617.	13. Ravaioli et al. 2020
11.	Carlsson S, Sandin F, Fall K, Lambe M, Adolfsson J, Stattin P, et al. Risk of suicide in men with low-risk prostate cancer. Eur J Cancer. 2013;49:1588-99.	14. Brunckhorst et al. 2021
12.	Crocetti, E., Arniani, S., Acciai, S., Barchielli, A., & Buiatti, E. (1998). High suicide mortality soon after diagnosis among cancer patients in central Italy. British Journal of Cancer, 77(7), 1194–1196. doi:10.1038/bjc.1998.199	15. Amiri and Behnezhad 2020<br>16. Du et al. 2020
13.	Dai, W.J., Gao, Y., Zhu, J.Y., 2008. Analysis of causes of suicide in cancer patients and nursing countermeasures. Shanghai Nurs. 8, 28–30.	17. Du et al. 2020
14.	Dalela D, Krishna N, Okwara J, Preston MA, Abdollah F, Choueiri TK, et al. Suicide and accidental deaths among patients with non-metastatic prostate cancer. BJUI Int. 2016;118:286-97.	18. Brunckhorst et al. 2021
15.	Dormer, N. R., McCaul, K. A., & Kristjanson, L. J. (2008). Risk of suicide in cancer patients in Western Australia, 1981-2002. The Medical Journal of Australia, 188(3), 140–143.	19. Amiri and Behnezhad 2020<br>20. Du et al. 2020<br>21. Ravaioli et al. 2020
16.	Dulskas, A., Patasius, A., Kaceniene, A., Urbonas, V., Smailyte, G., 2019. Suicide risk among colorectal cancer patients in Lithuania. Int. J. Colorectal Dis. 34, 555–558.	22. Du et al. 2020

17.	Fall, K., Fang, F., Mucci, L. A., Ye, W., Andren, O., Johansson, J.-E., … Valdimarsdottir, U. (2009). Immediate risk for cardiovascular events and suicide following a prostate cancer diagnosis: prospective cohort study. PLoS Medicine, 6(12), e1000197. doi:10.1371/journal.pmed.1000197	23. Amiri and Behnezhad 2020<br>24. Brunckhorst et al. 2021<br>25. Du et al. 2020
18.	Fang F, Keating NL, Mucci LA, Adami HO, Stampfer MJ, Valdimarsdóttir U, et al. Immediate risk of suicide and cardiovascular death after a prostate cancer diagnosis: Cohort study in the United States. J Natl Cancer Inst. 2010;102:307-14.	26. Brunckhorst et al. 2021
19.	Fang, F., Fall, K., Mittleman, M.A., Spar´en, P., Ye, W., Adami, H.-O., Valdimarsdottir, ´ U., 2012. Suicide and cardiovascular death after a cancer diagnosis. N. Engl. J. Med. 366 (14), 1310–1318.	27. Qin et al. 2022
20.	Guo, C., Zheng, W., Zhu, W., Yu, S., Ding, Y., Wu, Q., Tang, Q., Lu, C., 2019. Risk factors associated with suicide among kidney cancer patients: a surveillance, epidemiology, and end results analysis. Cancer Med. 8, 5386–5396.	28. Du et al. 2020
21.	Hem, E., Loge, J. H., Haldorsen, T., & Ekeberg, O. (2004). Suicide risk in cancer patients from 1960 to 1999. Journal of Clinical Oncology, 22(20), 4209–4216. doi:10.1200/JCO.2004. 02.052	29. Amiri and Behnezhad 2020<br>30. Brunckhorst et al. 2021<br>31. Du et al. 2020<br>32. Hofmann et al. 2023<br>33. Ravaioli et al. 2020
22.	Henson, K.E., Brock, R., Charnock, J., Wickramasinghe, B., Will, O., Pitman, A., 2019. Risk of suicide after cancer diagnosis in England. JAMA Psychiatry 76, 51–60.	34. Du et al. 2020<br>35. Hofmann et al. 2023
23.	Innos, K., Rahu, K., Rahu, M., & Baburin, A. (2003). Suicides among cancer patients in Estonia: a population-based study. European Journal of Cancer, 39(15), 2223–2228.	36. Amiri and Behnezhad 2020<br>37. Du et al. 2020<br>38. Hofmann et al. 2023
24.	Kaceniene, A.; Krilaviciute, A.; Kazlauskiene, J.; Bulotiene, G.; Smailyte, G. Increasing suicide risk among cancer patients in Lithuania from 1993 to 2012, a cancer registry-based study. Eur. J. Cancer Prev. 2017, 26, S197–S203.	39. Hofmann et al. 2023<br>40. Ravaioli et al. 2020
25.	Klaassen Z, Jen RP, Dibianco JM, Reinstatler L, Li Q, Madi R, et al. Factors associated with suicide in patients with genitourinary malignancies. Cancer. 2015;121:1864-72.	41. Brunckhorst et al. 2021<br>42. Du et al. 2020
26.	Korhonen LM, Taskinen M, Rantanen M, et al. Suicides and deaths linked to risky health behavior in childhood cancer patients: a Nordic population-based register study. Cancer. 2019;125 (20):3631-3638. doi:10.1002/cncr.32373	43. Lee et al. 2023
27.	Levi, F., Bulliard, J. L., & La Vecchia, C. (1991). Suicide risk among incident cases of cancer in the Swiss Canton of Vaud. Oncology, 48(1), 44–47. doi:10.1159/000226893	44. Amiri and Behnezhad 2020<br>45. Du et al. 2020<br>46. Hofmann et al. 2023<br>47. Ravaioli et al. 2020

28.	Lin, H. C., Wu, C. H., & Lee, H. C. (2009). Risk factors for suicide following hospital discharge among cancer patients. Psychooncology, 18(10), 1038–1044. doi:10.1002/pon.1483	48. Amiri and Behnezhad 2020
29.	Lin, P.H., Liao, S.C., Chen, I.M., 2017. Impact of universal health coverage on suicide risk in newly diagnosed cancer patients: population-based cohort study from 1985 to 2007 in Taiwan. Psychooncology 26, 1852–1859.	49. Du et al. 2020<br>50. Hofmann et al. 2023<br>51. Ravaioli et al. 2020
30.	Llorente MD, Burke M, Gregory GR, Bosworth HB, Grambow SC, Horner RD, et al. Prostate cancer: a significant risk factor for latelife suicide. Am J Geriatr Psychiatry. 2005;13:195–201.	52. Amiri and Behnezhad 2020<br>53. Brunckhorst et al. 2021
31.	Louhivuori, K. A., & Hakama, M. (1979). Risk of suicide among cancer patients. American Journal of Epidemiology, 109(1), 59–65.	54. Amiri and Behnezhad 2020<br>55. Hofmann et al. 2023<br>56. Ravaioli et al. 2020
32.	Lu, D., Fall, K., Sparen, P., Ye, W., Adami, H. O., Valdimarsdottir, U., & Fang, F. (2013). Suicide and suicide attempt after a cancer diagnosis among young individuals. Annals of Oncology, 24(12), 3112–3117. doi:10.1093/annonc/ mdt415	57. Amiri and Behnezhad 2020
33.	Mahdi, H., Swensen, R.E., Munkarah, A.R., Chiang, S., Luhrs, K., Lockhart, D., Kumar, S., 2011. Suicide in women with gynecologic cancer. Gynecol. Oncol. 122, 344–349.	58. Du et al. 2020
34.	Miccinesi G, Crocetti E, Benvenuti A, Pacl: E. Suicide mortality is decreasing among cancer patients in Central Italy. Eur J Cancer 2004;40:53e7. https://doi.org/10.1016/j.ejca.2003.12.022.	59. Ravaioli et al. 2020
35.	Miller, M., Mogun, H., Azrael, D., Hempstead, K., & Solomon, D. H. (2008). Cancer and the risk of suicide in older Americans. Journal of Clinical Oncology, 26(29), 4720–4724. doi: 10.1200/JCO.2007.14.3990	60. Amiri and Behnezhad 2020
36.	Misono, S., Weiss, N. S., Fann, J. R., Redman, M., & Yueh, B. (2008). Incidence of suicide in persons with cancer. Journal of Clinical Oncology, 26(29), 4731–4738. doi:10.1200/JCO.2007.13. 8941	61. Amiri and Behnezhad 2020<br>62. Brunckhorst et al. 2021<br>63. Du et al. 2020<br>64. Ravaioli et al. 2020
37.	Muff Christensen, M. L., Yousaf, U., Engholm, G., & Storm, H. H. (2006). Increased suicide risk among Danish women with non-melanoma skin cancer, 1971-1999. European Journal of Cancer Prevention, 15(3), 266–268. doi: 10.1097/01.cej.0000195712.64448.ba	65. Amiri and Behnezhad 2020
38.	Nakash, O., Barchana, M., Liphshitz, I., KeinanBoker, L., & Levav, I. (2013). The effect of cancer on suicide in ethnic groups with a differential suicide risk. European Journal of Public Health, 23(1), 114–115. doi:10.1093/eurpub/ cks045	66. Amiri and Behnezhad 2020

39.	Nakash, O., Liphshitz, I., Keinan-Boker, L., & Levav, I. (2013). The effect of cancer on suicide among elderly Holocaust survivors. Suicide and Life-Threatening Behavior, 43(3), 290–295. doi: 10.1111/sltb.12015	67. Amiri and Behnezhad 2020
40.	Nathan PC, Nachman A, Sutradhar R, et al. Adverse mental health outcomes in a population-based cohort of survivors of childhood cancer. Cancer. 2018;124(9):2045-2057.	68. Lee et al. 2023
41.	Oberaigner, W., Sperner-Unterweger, B., Fiegl, M., Geiger-Gritsch, S., Haring, C., 2014. Increased suicide risk in cancer patients in Tyrol/Austria. Gen. Hosp. Psychiatry 36, 483–487.	69. Du et al. 2020<br>70. Hofmann et al. 2023<br>71. Ravaioli et al. 2020
42.	Osazuwa-Peters, N., Arnold, L.D., Loux, T.M., Varvares, M.A., Schootman, M., 2018. Factors associated with increased risk of suicide among survivors of head and neck cancer: a population-based analysis. Oral Oncol. 81, 29–34.	72. Du et al. 2020
43.	Peng, J.L., Zhang, X.L., Zhan, F.Q., Li, C.Y., Li, Y.J., 2005. Analysis of suicidal behaviors of the hospitalized late-stage cancer patients and safe care management policies. J. Trop. Med. 5, 549–551.	73. Du et al. 2020
44.	Pham, T.T., Talukder, A.M., Walsh, N.J., Lawson, A.G., Jones, A.J., Bishop, J.L., 2019. Clinical and epidemiological factors associated with suicide in colorectal cancer. Support. Care Cancer 27, 617–621.	74. Du et al. 2020
45.	Rahouma, M., Kamel, M., Abouarab, A., Eldessouki, I., Nasar, A., Harrison, S., Lee, B., Shostak, E., Morris, J., Stiles, B., Altorki, N.K., Port, J.L., 2018. Lung cancer patients have the highest malignancy-associated suicide rate in USA: a population-based analysis. Ecancermedicalscience 12, 859.	75. Du et al. 2020<br>76. Hofmann et al. 2023
46.	Ravaioli A, Crocetti E, Mancini S, Baldacchini F, Giuliani O, Vattiato R, Bucchi L, Falcini F. Suicide death among cancer patients: new data from northern Italy, systematic review of the last 22 years and meta-analysis. Eur J Cancer. 2020 Jan;125:104-113. doi: 10.1016/j.ejca.2019.08.019.<br><br>Note: This study was an observational study and subsequently, they added this study into a systematic review with meta-analysis (see objectives of this study).	77. Ravaioli et al. 2020
47.	Robinson, D., Renshaw, C., Okello, C., Moller, H., & Davies, E. A. (2009). Suicide in cancer patients in South East England from 1996 to 2005: a population-based study. British Journal of Cancer, 101(1), 198–201. doi:10.1038/ sj.bjc.6605110	78. Amiri and Behnezhad 2020<br>79. Du et al. 2020<br>80. Ravaioli et al. 2020

48.	Schairer, C., Brown, L.M., Chen, B.E., Howard, R., Lynch, C.F., Hall, P., Storm, H., Pukkala, E., Anderson, A., Kaijser, M., Andersson, M., Joensuu, H., Fossa, S.D., Ganz, P.A., Travis, L.B., 2006. Suicide after breast cancer: an international population-based study of 723,810 women. J. Natl. Cancer Inst. 98, 1416–1419.	81. Du et al. 2020
49.	Shen, J., Zhu, M., Li, S., Wang, Q., Wu, J., Li, Y., Wang, Q., Bian, X., Yang, L., Jiang, X., Xie, J., Lu, Y., Wang, K., Li, L.J., 2020. Incidence and risk factors for suicide death among Kaposi's sarcoma patients: a surveillance, epidemiology, and end results analysis. Med. Sci. Monit.: Int. Med. J. Exp. Clin. Res. 26, e920711.	82. Du et al. 2020
50.	Siracuse, B.L., Gorgy, G., Ruskin, J., Beebe, K.S., 2017. What is the incidence of suicide in patients with bone and soft tissue cancer?: Suicide and Sarcoma. Clin. Orthop. Relat. Res. 475, 1439–1445.	83. Du et al. 2020
51.	Smailyte, G., Jasilionis, D., Kaceniene, A., Krilaviciute, A., Ambrozaitiene, D., & Stankuniene, V. (2013). Suicides among cancer patients in Lithuania: A population-based census-linked study. Cancer Epidemiology, 37(5), 714–718. doi: doi:10.1016/j.canep.2013.05.009	84. Amiri and Behnezhad 2020<br>85. Brunckhorst et al. 2021<br>86. Du et al. 2020
52.	Smith DP, Calopedos R, Bang A, Yu XQ, Egger S, Chambers S, et al. Increased risk of suicide in New South Wales men with prostate cancer: Analysis of linked population-wide data. PLoS ONE. 2018;13.	87. Brunckhorst et al. 2021<br>88. Du et al. 2020
53.	Sun, L.M., Lin, C.L., Hsu, C.Y., Kao, C.H., 2018. Risk of suicide attempts among colorectal cancer patients: a nationwide population-based matched cohort study. Psycho Oncol. 27 (12), 2794–2801.	89. Qin et al. 2022
54.	Tanaka H, Tsukuma H, Masaoka T, Ajiki W, Koyama Y, Kinoshita N, Hasuo S, Oshima A. Suicide risk among cancer patients: experience at one medical center in Japan, 1978-1994. Jpn J Cancer Res. 1999 Aug;90(8):812-7. doi: 10.1111/j.1349-7006.1999.tb00820.x.	90. Amiri and Behnezhad 2020<br>91. Du et al. 2020<br>92. Hofmann et al. 2023
55.	Turaga, K.K., Malafa, M.P., Jacobsen, P.B., Schell, M.J., Sarr, M.G., 2011. Suicide in patients with pancreatic cancer. Cancer 117, 642–647.	93. Du et al. 2020
56.	Vyssoki, B., Gleiss, A., Rockett, I. R., Hackl, M., Leitner, B., Sonneck, G., & Kapusta, N. D. (2015). Suicide among 915,303 Austrian cancer patients: who is at risk?. J Affect Disord, 175, 287–291. doi:10.1016/j.jad.2015.01.028	94. Amiri and Behnezhad 2020<br>95. Brunckhorst et al. 2021<br>96. Du et al. 2020<br>97. Ravaioli et al. 2020
57.	Yamauchi, T., Inagaki, M., Yonemoto, N., Iwasaki, M., Inoue, M., Akechi, T., … Tsugane, S. (2014). Death by suicide and other externally caused injuries following a cancer	98. Amiri and Behnezhad 2020<br>99. Du et al. 2020

	diagnosis: the Japan Public Health Center-based Prospective Study. Psychooncology, 23(9), 1034–1041. doi: 10.1002/pon.3529	
58.	Yousaf U, Christensen ML, Engholm G, Storm HH. Suicides among Danish cancer patients 1971-1999. Br J Cancer. 2005 Mar 28;92(6):995-1000. doi: 10.1038/sj.bjc.6602424.	100. Amiri and Behnezhad 2020<br>101. Du et al. 2020
59.	Zaorsky, N.G., Zhang, Y., Tuanquin, L., Bluethmann, S.M., Park, H.S., Chinchilli, V.M., 2019. Suicide among cancer patients. Nat. Commun. 10, 207.	102. Du et al. 2020

Suicidal ideation

$$CCA = \frac{N-r}{rc-r} = \frac{21-21}{63-21} \quad \frac{0}{42} \quad = 0 = 0\%$$

Note: N is the total number of original studies (including duplicates) in the meta-analyses of interest (the sum of all checked boxes in the citation matrix). Furthermore, r is the number of original studies without accounting for duplicates. Finally, c is the number of systematic reviews included in the evidence matrix (k=3). CCA = corrected covered area.

**Number of studies without accounting for duplicates (r)**	**References of primary research**	**Systematic reviews where appear primary research including primary research duplicates (N)**
1.	Abdel-Rahman O. Socioeconomic predictors of suicide risk among cancer patients in the United States: a population-based study. Cancer Epidemiol 2019;63:101601.	1. Guo et al. 2021
2.	Bill-Axelson A, Garmo H, Lambe M, Bratt O, Adolfsson J, Nyberg U, et al. Suicide Risk in Men with Prostate-Specific Antigen-Detected Early Prostate Cancer: A Nationwide Population-Based Cohort Study from PCBaSe Sweden. Eur Urol. 2010;57:390-5.	2. Guo et al. 2018
3.	Carlsson S, Sandin F, Fall K, Lambe M, Adolfsson J, Stattin P, et al. Risk of suicide in men with low-risk prostate cancer. Eur J Cancer. 2013;49:1588-99.	3. Guo et al. 2018
4.	Chen Y-Z, Chiang P-K, Lin W-R, Chen M, Chow Y-C, Chiu AW, et al. The relationship between androgen deprivation therapy and depression symptoms in patients with prostate cancer. Aging Male. 2019.	4. Brunckhorst et al. 2021
5.	Choi JW, Park EC. Suicide risk after cancer diagnosis among older adults: a nationwide retrospective cohort study. J Geriatr Oncol 2019: pii: S1879-4068(19)30379−0.	5. Guo et al. 2021

6.	Dalela D, Krishna N, Okwara J, Preston MA, Abdollah F, Choueiri TK, et al. Suicide and accidental deaths among patients with non-metastatic prostate cancer. BJUI Int. 2016;118:286-97.	6. Guo et al. 2018
7.	Erlangsen A, Stenager E, Conwell Y. Physical diseases as predictors of suicide in older adults: a nationwide, register-based cohort study. Soc Psychiatry Psychiatr Epidemiol 2015;50:1427–39.	7. Guo et al. 2021
8.	Fall, K., Fang, F., Mucci, L. A., Ye, W., Andren, O., Johansson, J.-E., … Valdimarsdottir, U. (2009). Immediate risk for cardiovascular events and suicide following a prostate cancer diagnosis: prospective cohort study. PLoS Medicine, 6(12), e1000197. doi:10.1371/journal.pmed.1000197	8. Guo et al. 2018
9.	Fang F, Keating NL, Mucci LA, Adami HO, Stampfer MJ, Valdimarsdóttir U, et al. Immediate risk of suicide and cardiovascular death after a prostate cancer diagnosis: Cohort study in the United States. J Natl Cancer Inst. 2010;102:307-14.	9. Guo et al. 2018
10.	Juurlink DN, Herrmann N, Szalai JP, Kopp A, Redelmeier DA. Medical illness and the risk of suicide in the elderly. Arch Intern Med. 2004;164:1179–84.	10. Guo et al. 2018
11.	Klaassen Z, Goldberg H, Chandrasekar T, Arora K, Sayyid RK, Hamilton RJ, et al. Changing trends for suicidal death in patients with bladder cancer: A 40+ year population-level analysis. Clin Genitourin Cancer 2018;16:206–12	11. Guo et al. 2021
12.	Klaassen Z, Wallis CJD, Chandrasekar T, Goldberg H, Sayyid RK, Williams SB, et al. Cancer diagnosis and risk of suicide after accounting for prediagnosis psychiatric care: a matched-cohort study of patients with incident solid-organ malignancies. Cancer 2019; 125:2886–95.	12. Guo et al. 2021
13.	Lehto US, Helander S, Taari K, Aromaa A. Patient experiences at diagnosis and psychological well-being in prostate cancer: A Finnish national survey. Eur J Oncol Nurs. 2015;19:220-9.	13. Brunckhorst et al. 2021
14.	Lehuluante A, Fransson P. Are there specific health-related factors that can accentuate the risk of suicide among men with prostate cancer? Support Care Cancer. 2014;22:1673-8.	14. Brunckhorst et al. 2021
15.	Llorente MD, Burke M, Gregory GR, Bosworth HB, Grambow SC, Horner RD, et al. Prostate cancer: a significant risk factor for latelife suicide. Am J Geriatr Psychiatry. 2005;13:195–201.	15. Guo et al. 2018

16.	Louda M, Vališ M, Šplíchalová J, Pacovský J, Khaled B, Podhola M, et al. Psychosocial implications and the duality of life outcomes for patients with prostate carcinoma after bilateral orchiectomy. Neuro Endocrinol Lett. 2012;33:761-4.	16. Brunckhorst et al. 2021
17.	Perry LM, Hoerger M, Silberstein J, Sartor O, Duberstein P. Understanding the distressed prostate cancer patient: Role of personality. Psycho-Oncology. 2018;27:810-6.	17. Brunckhorst et al. 2021
18.	Recklitis CJ, Zhou ES, Zwemer EK, Hu JC, Kantoff PW. Suicidal ideation in prostate cancer survivors: Understanding the role of physical and psychological health outcomes. Cancer. 2014;120:3393-400.	18. Brunckhorst et al. 2021
19.	Rice SM, Oliffe JL, Kelly MT, Cormie P, Chambers S, Ogrodniczuk JS, et al. Depression and Prostate Cancer: Examining Comorbidity and Male-Specific Symptoms. Am J Mens Health. 2018;12:1864-72.	19. Brunckhorst et al. 2021
20.	Smith DP, Bang A, Egger S, Yu XQ, Egger S, Chambers S, et al. Risk of suicide after a prostate cancer diagnosis: a populationwide study in New South Wales (NSW) Australia. BJU Int. 2015;116:22.	20. Guo et al. 2018
21.	Zhou ES, Hu JC, Kantoff PW, Recklitis CJ. Identifying suicidal symptoms in prostate cancer survivors using brief self-report. J Cancer Surviv. 2015;9:59-67.	21. Brunckhorst et al. 2021
