# Supplementary material for: Suicidal Ideation, Suicide Attempts, and Suicide Mortality in Cancer: An Overview of Systematic Reviews with Meta-Analysis
Source: Cancers (Basel). 2025 May 27;17(11):1788. doi: 10.3390/cancers17111788 (PMC12153619; doi:10.3390/cancers17111788)
Supplement: Supplementary file 1 [file cancers-17-01788-s001.zip › Suppl File 7 Overlap Prostate Cancer.pdf]

Supplementary file 7. Matrices of evidence and the corrected covered area (CCA) calculations by specific cancer sites.

Suicide mortality in reviews focused on prostate cancer.

$$CCA = \frac{N-r}{rc-r} = \frac{27-15}{45-15} \quad \frac{12}{30} \quad = 0.4 = 40\%$$

Note: N is the total number of original studies (including duplicates) in the meta-analyses of interest (the sum of all checked boxes in the citation matrix). Furthermore, r is the number of original studies without accounting for duplicates. Finally, c is the number of systematic reviews included in the evidence matrix (k=3). CCA = corrected covered area.

Number of studies without accounting for duplicates (r)	References of primary research	Systematic reviews where appear primary research including primary research duplicates (N)
1.	Bill-Axelson A, Garmo H, Lambe M, Bratt O, Adolfsson J, Nyberg U, et al. Suicide Risk in Men with Prostate-Specific Antigen-Detected Early Prostate Cancer: A Nationwide Population-Based Cohort Study from PCBaSe Sweden. Eur Urol. 2010;57:390-5.	1. Brunckhorst et al. 2021<br>2. Guo et al. 2018
2.	Carlsson S, Sandin F, Fall K, Lambe M, Adolfsson J, Stattin P, et al. Risk of suicide in men with low-risk prostate cancer. Eur J Cancer. 2013;49:1588-99.	3. Brunckhorst et al. 2021<br>4. Guo et al. 2018
3.	Dalela D, Krishna N, Okwara J, Preston MA, Abdollah F, Choueiri TK, et al. Suicide and accidental deaths among patients with non-metastatic prostate cancer. BJUI Int. 2016;118:286-97.	5. Brunckhorst et al. 2021<br>6. Guo et al. 2018
4.	Fall, K., Fang, F., Mucci, L. A., Ye, W., Andren, O., Johansson, J.-E., … Valdimarsdottir, U. (2009). Immediate risk for cardiovascular events and suicide following a prostate cancer diagnosis: prospective cohort study. PLoS Medicine, 6(12), e1000197. doi:10.1371/journal.pmed.1000197	7. Amiri and Behnezhad 2020<br>8. Brunckhorst et al. 2021<br>9. Guo et al. 2018

5.	Fang F, Keating NL, Mucci LA, Adami HO, Stampfer MJ, Valdimarsdóttir U, et al. Immediate risk of suicide and cardiovascular death after a prostate cancer diagnosis: Cohort study in the United States. J Natl Cancer Inst. 2010;102:307-14.	10. Brunckhorst et al. 2021<br>11. Guo et al. 2018
6.	Hem, E., Loge, J. H., Haldorsen, T., & Ekeberg, O. (2004). Suicide risk in cancer patients from 1960 to 1999. Journal of Clinical Oncology, 22(20), 4209–4216. doi:10.1200/JCO.2004. 02.052	12. Amiri and Behnezhad 2020<br>13. Brunckhorst et al. 2021
7.	Innos, K., Rahu, K., Rahu, M., & Baburin, A. (2003). Suicides among cancer patients in Estonia: a population-based study. European Journal of Cancer, 39(15), 2223–2228	14. Amiri and Behnezhad 2020
8.	Juurlink DN, Herrmann N, Szalai JP, Kopp A, Redelmeier DA. Medical illness and the risk of suicide in the elderly. Arch Intern Med. 2004;164:1179–84.	15. Guo et al. 2018
9.	Klaassen Z, Jen RP, Dibianco JM, Reinstatler L, Li Q, Madi R, et al. Factors associated with suicide in patients with genitourinary malignancies. Cancer. 2015;121:1864-72.	16. Brunckhorst et al. 2021
10.	Llorente MD, Burke M, Gregory GR, Bosworth HB, Grambow SC, Horner RD, et al. Prostate cancer: a significant risk factor for latelife suicide. Am J Geriatr Psychiatry. 2005;13:195–201.	17. Amiri and Behnezhad 2020<br>18. Brunckhorst et al. 2021<br>19. Guo et al. 2018
11.	Misono, S., Weiss, N. S., Fann, J. R., Redman, M., & Yueh, B. (2008). Incidence of suicide in persons with cancer. Journal of Clinical Oncology, 26(29), 4731–4738. doi:10.1200/JCO.2007.13. 8941	20. Amiri and Behnezhad 2020<br>21. Brunckhorst et al. 2021
12.	Smailyte, G., Jasilionis, D., Kaceniene, A., Krilaviciute, A., Ambrozaitiene, D., & Stankuniene, V. (2013). Suicides among cancer patients in Lithuania: A population-based census-linked study. Cancer Epidemiology, 37(5), 714–718. doi: doi:10.1016/j.canep.2013.05.009	22. Amiri and Behnezhad 2020<br>23. Brunckhorst et al. 2021
13.	Smith DP, Bang A, Egger S, Yu XQ, Egger S, Chambers S, et al. Risk of suicide after a prostate cancer diagnosis: a populationwide study in New South Wales (NSW) Australia. BJU Int. 2015;116:22.	24. Guo et al. 2018
14.	Smith DP, Calopedos R, Bang A, Yu XQ, Egger S, Chambers S, et al. Increased risk of suicide in New South Wales men with prostate cancer: Analysis of linked population-wide data. PLoS ONE. 2018;13.	25. Brunckhorst et al. 2021
15.	Vyssoki, B., Gleiss, A., Rockett, I. R., Hackl, M., Leitner, B., Sonneck, G., & Kapusta, N. D. (2015). Suicide among 915,303 Austrian cancer patients: who is at risk?. J Affect Disord, 175, 287–291. doi:10.1016/j.jad.2015.01.028	26. Amiri and Behnezhad 2020<br>27. Brunckhorst et al. 2021

Suicidal ideation in reviews focused on prostate cancer.

$$CCA = \frac{N-r}{rc-r} = \frac{16-16}{32-16} \quad \frac{0}{16} \quad = 0 = 0\%$$

Note: N is the total number of original studies (including duplicates) in the meta-analyses of interest (the sum of all checked boxes in the citation matrix). Furthermore, r is the number of original studies without accounting for duplicates. Finally, c is the number of systematic reviews included in the evidence matrix (k=2). CCA = corrected covered area.

Number of studies without accounting for duplicates (r)	References of primary research	Systematic reviews where appear primary research including primary research duplicates (N)
1.	Bill-Axelson A, Garmo H, Lambe M, Bratt O, Adolfsson J, Nyberg U, et al. Suicide Risk in Men with Prostate-Specific Antigen-Detected Early Prostate Cancer: A Nationwide Population-Based Cohort Study from PCBaSe Sweden. Eur Urol. 2010;57:390-5.	1. Guo et al. 2018
2.	Carlsson S, Sandin F, Fall K, Lambe M, Adolfsson J, Stattin P, et al. Risk of suicide in men with low-risk prostate cancer. Eur J Cancer. 2013;49:1588-99.	2. Guo et al. 2018
3.	Chen Y-Z, Chiang P-K, Lin W-R, Chen M, Chow Y-C, Chiu AW, et al. The relationship between androgen deprivation therapy and depression symptoms in patients with prostate cancer. Aging Male. 2019.	3. Brunckhorst et al. 2021
4.	Dalela D, Krishna N, Okwara J, Preston MA, Abdollah F, Choueiri TK, et al. Suicide and accidental deaths among patients with non-metastatic prostate cancer. BJUI Int. 2016;118:286-97.	4. Guo et al. 2018
5.	Fall, K., Fang, F., Mucci, L. A., Ye, W., Andren, O., Johansson, J.-E., … Valdimarsdottir, U. (2009). Immediate risk for cardiovascular events and suicide following a prostate cancer	5. Guo et al. 2018

	diagnosis: prospective cohort study. PLoS Medicine, 6(12), e1000197. doi:10.1371/journal.pmed.1000197	
6.	Fang F, Keating NL, Mucci LA, Adami HO, Stampfer MJ, Valdimarsdóttir U, et al. Immediate risk of suicide and cardiovascular death after a prostate cancer diagnosis: Cohort study in the United States. J Natl Cancer Inst. 2010;102:307-14.	6. Guo et al. 2018
7.	Juurlink DN, Herrmann N, Szalai JP, Kopp A, Redelmeier DA. Medical illness and the risk of suicide in the elderly. Arch Intern Med. 2004;164:1179–84.	7. Guo et al. 2018
8.	Lehto US, Helander S, Taari K, Aromaa A. Patient experiences at diagnosis and psychological well-being in prostate cancer: A Finnish national survey. Eur J Oncol Nurs. 2015;19:220-9.	8. Brunckhorst et al. 2021
9.	Lehuluante A, Fransson P. Are there specific health-related factors that can accentuate the risk of suicide among men with prostate cancer? Support Care Cancer. 2014;22:1673-8.	9. Brunckhorst et al. 2021
10.	Llorente MD, Burke M, Gregory GR, Bosworth HB, Grambow SC, Horner RD, et al. Prostate cancer: a significant risk factor for latelife suicide. Am J Geriatr Psychiatry. 2005;13:195–201.	10. Guo et al. 2018
11.	Louda M, Vališ M, Šplíchalová J, Pacovský J, Khaled B, Podhola M, et al. Psychosocial implications and the duality of life outcomes for patients with prostate carcinoma after bilateral orchiectomy. Neuro Endocrinol Lett. 2012;33:761-4.	11. Brunckhorst et al. 2021
12.	Perry LM, Hoerger M, Silberstein J, Sartor O, Duberstein P. Understanding the distressed prostate cancer patient: Role of personality. Psycho-Oncology. 2018;27:810-6.	12. Brunckhorst et al. 2021
13.	Recklitis CJ, Zhou ES, Zwemer EK, Hu JC, Kantoff PW. Suicidal ideation in prostate cancer survivors: Understanding the role of physical and psychological health outcomes. Cancer. 2014;120:3393-400.	13. Brunckhorst et al. 2021
14.	Rice SM, Oliffe JL, Kelly MT, Cormie P, Chambers S, Ogrodniczuk JS, et al. Depression and Prostate Cancer: Examining Comorbidity and Male-Specific Symptoms. Am J Mens Health. 2018;12:1864-72.	14. Brunckhorst et al. 2021
15.	Smith DP, Bang A, Egger S, Yu XQ, Egger S, Chambers S, et al. Risk of suicide after a prostate cancer diagnosis: a populationwide study in New South Wales (NSW) Australia. BJU Int. 2015;116:22.	15. Guo et al. 2018
16.	Zhou ES, Hu JC, Kantoff PW, Recklitis CJ. Identifying suicidal symptoms in prostate cancer survivors using brief self-report. J Cancer Surviv. 2015;9:59-67.	16. Brunckhorst et al. 2021

Suicide mortality in reviews focused on lung/bronchus/trachea cancer.

$$CCA = \frac{N-r}{rc-r} = \frac{20-16}{32-16} \frac{4}{16} = 0.25 = 25\%$$

Note: N is the total number of original studies (including duplicates) in the meta-analyses of interest (the sum of all checked boxes in the citation matrix). Furthermore, r is the number of original studies without accounting for duplicates. Finally, c is the number of systematic reviews included in the evidence matrix (k=2). CCA = corrected covered area.

**Number of studies without accounting for duplicates (r)**	**References of primary research**	**Systematic reviews where appear primary research including primary research duplicates (N)**
1.	Ahn, E., Shin, D.W., Cho, S.I., Park, S., Won, Y.J., Yun, Y.H., 2010. Suicide rates and risk factors among Korean cancer patients, 1993-2005. Cancer Epidemiol. Biomark. Prevent.: Publ. Am. Assoc. Cancer Res. Cosponsored Am. Soc. Prevent. Oncol. 19, 2097–2105.	1. Hofmann et al. 2023
2.	Ahn, M. H., Park, S., Lee, H. B., Ramsey, C. M., Na, R., Kim, S. O., … Hong, J. P. (2015). Suicide in cancer patients within the first year of diagnosis. Psychooncology, 24(5), 601–607. doi: 10.1002/pon.3705	2. Amiri and Behnezhad 2020
3.	Allebeck, P.; Bolund, C. Suicides and suicide attempts in cancer patients. Psychol. Med. 1991, 21, 979–984.	3. Hofmann et al. 2023
4.	Hem, E., Loge, J. H., Haldorsen, T., & Ekeberg, O. (2004). Suicide risk in cancer patients from 1960 to 1999. Journal of Clinical Oncology, 22(20), 4209–4216. doi:10.1200/JCO.2004. 02.052	4. Amiri and Behnezhad 2020<br>5. Hofmann et al. 2023
5.	Henson, K.E., Brock, R., Charnock, J., Wickramasinghe, B., Will, O., Pitman, A., 2019. Risk of suicide after cancer diagnosis in England. JAMA Psychiatry 76, 51–60.	6. Hofmann et al. 2023
6.	Innos, K., Rahu, K., Rahu, M., & Baburin, A. (2003). Suicides among cancer patients in Estonia: a population-based study. European Journal of Cancer, 39(15), 2223–2228.	7. Amiri and Behnezhad 2020<br>8. Hofmann et al. 2023

7.	Kaceniene, A.; Krilaviciute, A.; Kazlauskiene, J.; Bulotiene, G.; Smailyte, G. Increasing suicide risk among cancer patients in Lithuania from 1993 to 2012, a cancer registry-based study. Eur. J. Cancer Prev. 2017, 26, S197–S203.	9. Hofmann et al. 2023
8.	Levi, F., Bulliard, J. L., & La Vecchia, C. (1991). Suicide risk among incident cases of cancer in the Swiss Canton of Vaud. Oncology, 48(1), 44–47. doi:10.1159/000226893	10. Amiri and Behnezhad 2020<br>11. Hofmann et al. 2023
9.	Lin, P.H., Liao, S.C., Chen, I.M., 2017. Impact of universal health coverage on suicide risk in newly diagnosed cancer patients: population-based cohort study from 1985 to 2007 in Taiwan. Psychooncology 26, 1852–1859.	12. Hofmann et al. 2023
10.	Louhivuori, K. A., & Hakama, M. (1979). Risk of suicide among cancer patients. American Journal of Epidemiology, 109(1), 59–65.	13. Hofmann et al. 2023
11.	Misono, S., Weiss, N. S., Fann, J. R., Redman, M., & Yueh, B. (2008). Incidence of suicide in persons with cancer. Journal of Clinical Oncology, 26(29), 4731–4738. doi:10.1200/JCO.2007.13. 8941	14. Amiri and Behnezhad 2020
12.	Oberaigner, W., Sperner-Unterweger, B., Fiegl, M., Geiger-Gritsch, S., Haring, C., 2014. Increased suicide risk in cancer patients in Tyrol/Austria. Gen. Hosp. Psychiatry 36, 483–487.	15. Hofmann et al. 2023
13.	Rahouma, M., Kamel, M., Abouarab, A., Eldessouki, I., Nasar, A., Harrison, S., Lee, B., Shostak, E., Morris, J., Stiles, B., Altorki, N.K., Port, J.L., 2018. Lung cancer patients have the highest malignancy-associated suicide rate in USA: a population-based analysis. Ecancermedicalscience 12, 859.	16. Hofmann et al. 2023
14.	Smailyte, G., Jasilionis, D., Kaceniene, A., Krilaviciute, A., Ambrozaitiene, D., & Stankuniene, V. (2013). Suicides among cancer patients in Lithuania: A population-based census-linked study. Cancer Epidemiology, 37(5), 714–718. doi: doi:10.1016/j.canep.2013.05.009	17. Amiri and Behnezhad 2020
15.	Tanaka H, Tsukuma H, Masaoka T, Ajiki W, Koyama Y, Kinoshita N, Hasuo S, Oshima A. Suicide risk among cancer patients: experience at one medical center in Japan, 1978-1994. Jpn J Cancer Res. 1999 Aug;90(8):812-7. doi: 10.1111/j.1349-7006.1999.tb00820.x.	18. Amiri and Behnezhad 2020<br>19. Hofmann et al. 2023
16.	Vyssoki, B., Gleiss, A., Rockett, I. R., Hackl, M., Leitner, B., Sonneck, G., & Kapusta, N. D. (2015). Suicide among 915,303 Austrian cancer patients: who is at risk?. J Affect Disord, 175, 287–291. doi:10.1016/j.jad.2015.01.028	20. Amiri and Behnezhad 2020
