# Supplementary figures and images for: Suicidal Ideation, Suicide Attempts, and Suicide Mortality in Cancer: An Overview of Systematic Reviews with Meta-Analysis
Source: Cancers (Basel). 2025 May 27;17(11):1788. doi: 10.3390/cancers17111788 (PMC12153619; doi:10.3390/cancers17111788)

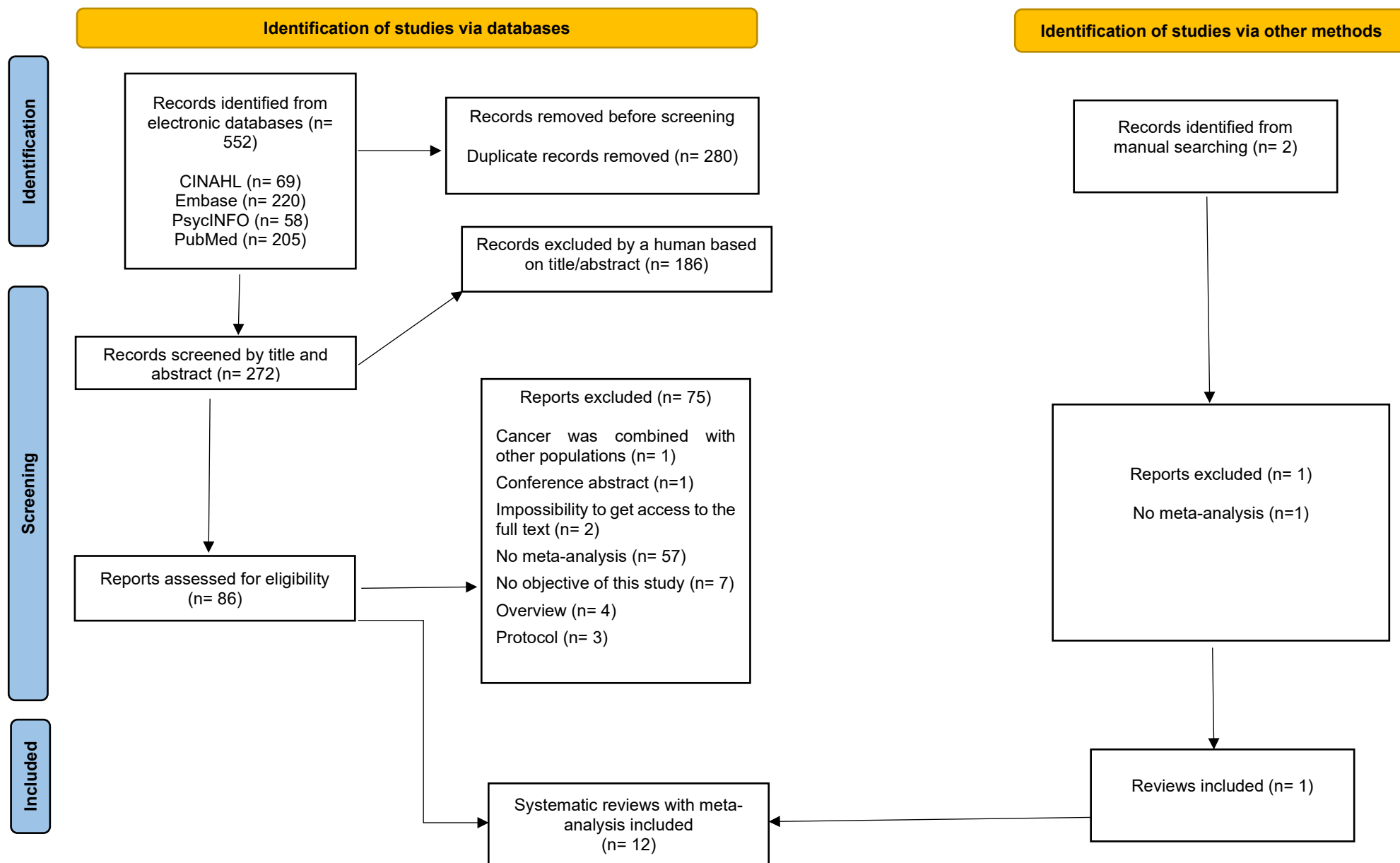

**Supplementary file 3.** Flow Diagram: study selection process.

Supplement: Supplementary file 1 [file cancers-17-01788-s001.zip › Suppl File 3 Flow Diagram.pdf]

### Overall Overlap (CCA) for each type of suicide

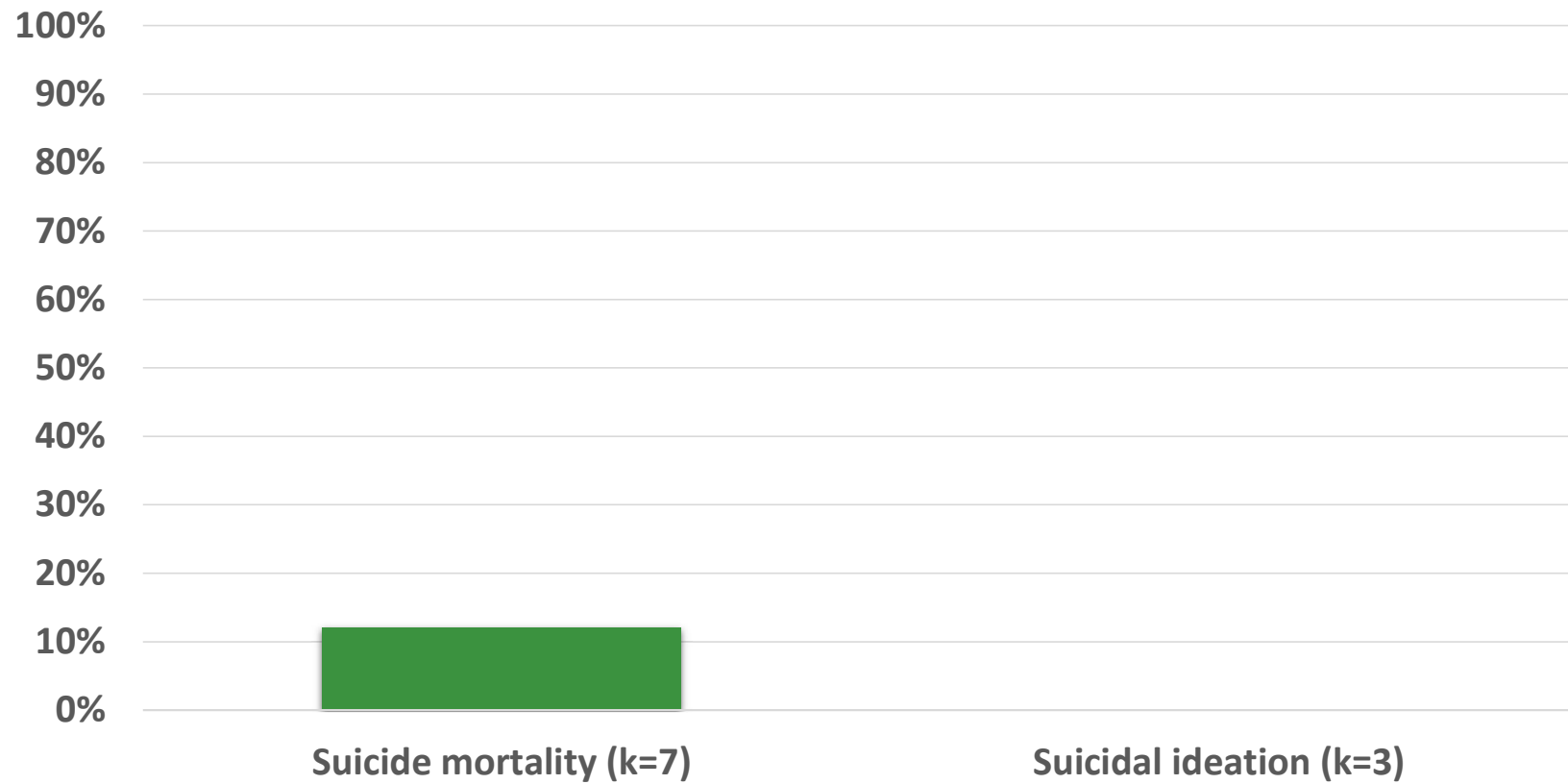

Supplement: Supplementary file 1 [file cancers-17-01788-s001.zip › Suppl File 6 Bar plot overall overlap.pdf]

## Overlap (CCA) for each type of suicide regarding cancer site

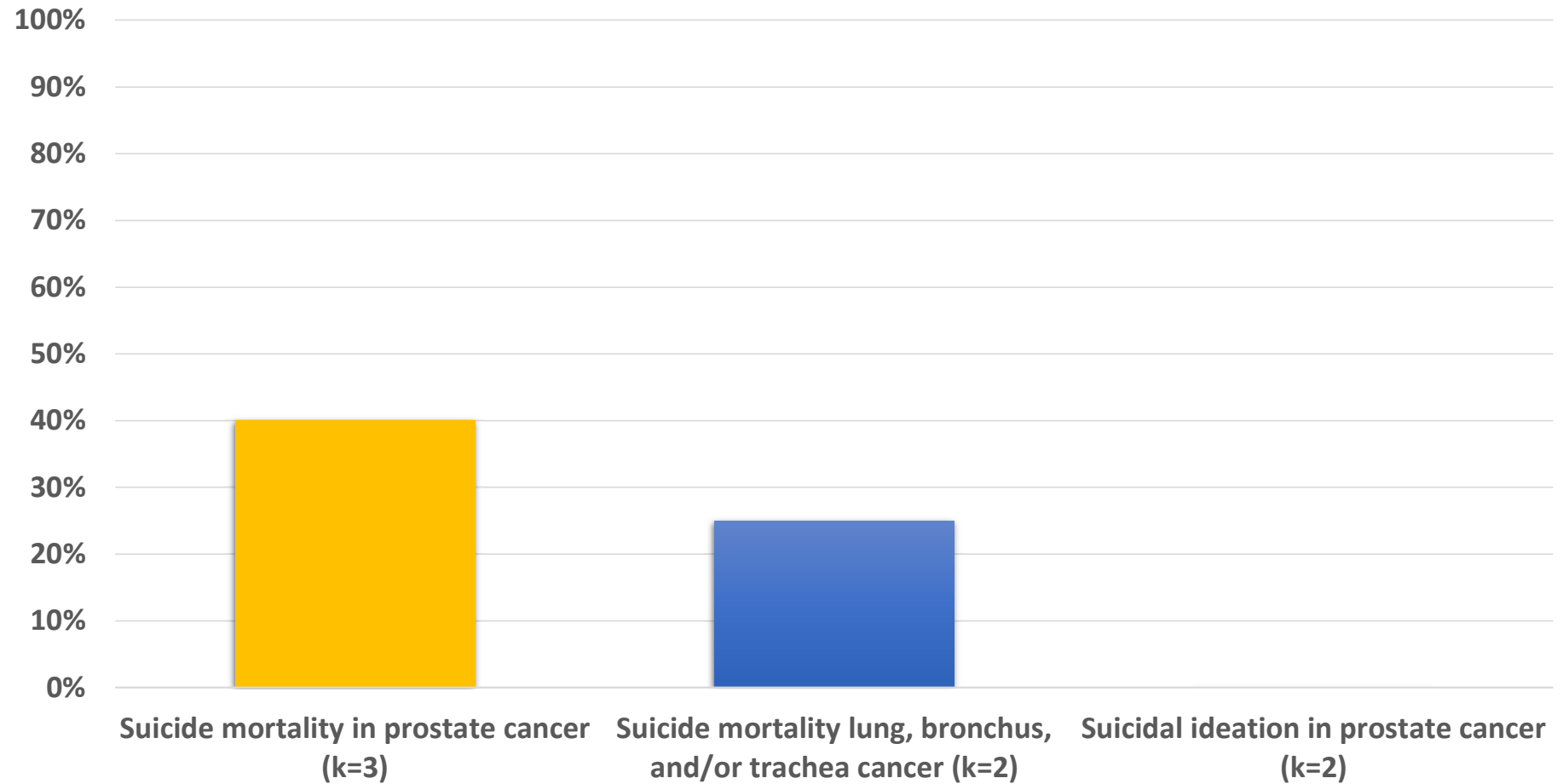

Supplement: Supplementary file 1 [file cancers-17-01788-s001.zip › Suppl File 8 Bar plot overlap cancer sites.pdf]
